# Niosomal l-carnitine and quercetin improve sperm quality and testicular function in atrazine-induced reproductive toxicity in rats

**DOI:** 10.1038/s41598-026-58143-4

**Published:** 2026-06-22

**Authors:** Omniya E. Azmi, Ahmed Abdel-Wahab, Mootaz A. M. Abdel-Rahman, Amr Gamal, Marwa A. Ibrahim, Osama Mohamed Ahmed, Abdel‑Razik H. Abdel‑Razik, Safwat Ali, A. A. M. El-Gendy

**Affiliations:** 1 Department of Pharmacology, Faculty of Veterinary Medicine, Minia University, El-Minia, 61519 Egypt; 2 Physiology Department, Faculty of Veterinary Medicine, Minia University, El-Minia, 61519 Egypt; 3 Department of Behavior, Management and Development of Animal Wealth, Faculty of Veterinary Medicine, Minia University, El-Minia, 61519 Egypt; 4 Department of Pharmaceutics and Industrial Pharmacy, Faculty of Pharmacy, Beni-Suef University, Beni-Suef, Egypt; 5 Department of Biochemistry and Molecular Biology, Faculty of Veterinary Medicine, Cairo University, Giza, 12211 Egypt; 6 Physiology Division, Department of Zoology, Faculty of Science, Beni-Suef University, P.O. Box 62521, Beni-Suef, 62521 Egypt; 7 Department of Histology, Faculty of Veterinary Medicine, Beni-Suef University, Beni-Suef, 62511 Egypt; 8 Department of Anatomy and Embryology, Faculty of Veterinary Medicine, Minia University, El-Minia, 61519 Egypt; 9 Department of Pharmacology, Faculty of Veterinary Medicine, Beni-Suef University, 62511 Beni-Suef, Egypt

**Keywords:** L-carnitine, Quercetin, Niosomal formulations, Atrazine, Testes, Biochemistry, Drug discovery, Physiology

## Abstract

Atrazine (ATZ), a frequently employed herbicide, is classified as one of the environmental pollutants that play a pivotal role in progression of male infertility. L-carnitine (LC) and quercetin (QT) possess antioxidant properties, rendering them viable additional treatments for male infertility. Additionally, specialized drug delivery systems, such as niosomes, enhance the distribution of hydrophilic pharmaceuticals. This study aimed to investigate the ameliorative potential of LC and QT, administered in conventional and niosomal formulations, against ATZ-induced testicular dysfunction in adult male albino rats. Thirty rats were randomly assigned to six experimental groups: control, ATZ, ATZ + LC, ATZ + LC-loaded niosomes (LCLN), ATZ + QT, and ATZ + QT-loaded niosomes (QTLN). Treatments were administered orally for 56 consecutive days. Biochemical analyses, sperm evaluations, gene expression assessments, and histopathological measures were conducted. ATZ exposure significantly decreased absolute and relative seminal vesicle weights, impaired sperm motility, viability, morphology and count, and reduced circulating testosterone, follicle-stimulating hormone (FSH), and luteinizing hormone (LH) concentrations. Moreover, ATZ markedly elevated malondialdehyde levels, suppressed the activities of endogenous antioxidant enzymes (superoxide dismutase, catalase, glutathione peroxidase), and downregulated the transcription of key steroidogenic genes (*HSD3B*,* StAR* and *CYP11A1*). Histopathological assessment further revealed pronounced degenerative changes in the testes, epididymis, seminal vesicles, and prostate. Co-administration of LC or QT, particularly in niosomal formulations, significantly attenuated these deleterious effects. They restored sperm quality, re-established redox homeostasis, normalized steroidogenic gene expression, and preserved the structural integrity of reproductive tissues. In conclusion, niosomal formulations of LC and QT confer superior protective efficacy against ATZ-induced testicular toxicity compared to their conventional counterparts, underscoring their therapeutic potential as targeted interventions against environmental toxicants.

**Supplementary Information:**

The online version contains supplementary material available at 10.1038/s41598-026-58143-4.

## Introduction

Male infertility is an escalating global issue, with environmental pollutants such as pesticides significantly contributing to its rising incidence^1^. Atrazine (ATZ), a commonly utilized herbicide, is among these contaminants. Based on extensive research, the primary sources of ATZ contamination in the environment are its agricultural application and subsequent transport mechanisms^2^. Following its use as a herbicide, ATZ exhibits high mobility in soil, resulting in its widespread presence in groundwater and surface water through leaching and runoff^2,3^. A significant pathway for dispersion is its sensitivity to running water and precipitation, which facilitates its movement into tributaries, lagoons, and other aquatic channels, with an estimated 1 to 6% of applied herbicides reaching these environments^4^. Furthermore, some ATZ particles can become airborne via volatilization and be redistributed through atmospheric deposition by rain^4^. This environmental persistence is exacerbated by its slow decomposition rate, with a half-life estimated between 95 and 350 days in the water column^5^. These combined factors result in the contamination of freshwater resources, which subsequently acts as a critical vector for exposure in both wildlife and humans through the ingestion of contaminated water and the food chain^6^.

Atrazine has been identified as a potent endocrine disruptor that adversely affects male reproductive health^7^. ATZ exposure has been linked to oxidative stress, hormonal imbalances, and impaired spermatogenesis, leading to reduced sperm quality and fertility^3^. ATZ was observed to downregulate key steroidogenic enzymes, including *3β-HSD* and *Cyp17a1*, and disrupt the expression of genes critical for testosterone production, such as *StAR*^8–10^. The consequent impact on spermatogenesis is profound, resulting in significantly reduced sperm count, motility, and increased sperm abnormalities and DNA fragmentation, which collectively impair fertilization potential and embryo development^11^. A primary mechanism underlying this toxicity is oxidative stress, evidenced by increased lipid peroxidation (malondialdehyde “MDA”) and depletion of antioxidant defenses (superoxide dismutase “SOD”) within reproductive tissues, leading to inflammation and cellular damage^7^. Given its persistence in groundwater and its documented toxic effects, there is an urgent need for protective agents that can mitigate ATZ-induced reproductive damage.

L-carnitine (LC) and quercetin (QT) are two naturally occurring compounds with well-established antioxidant and anti-inflammatory properties. LC plays a crucial role in energy metabolism and has been shown to improve sperm motility, viability, and oxidative defense mechanisms^12^. L-carnitine plays a crucial role in male fertility, primarily due to its high concentration in the epididymis and spermatozoa (approximately 2000 times higher than in plasma) and its essential function in transporting fatty acids for mitochondrial energy production^13^. Beyond these benefits, LC acts at a genetic level by lowering the sperm DNA fragmentation index and may serve as an epigenetic regulator, while also supporting Sertoli cell function and spermatogenesis^14,15^. Clinically, co-supplementation with acetyl-L-carnitine has proven effective in improving pregnancy rates for men with idiopathic oligoasthenoteratozoospermia^16^.

Similarly, QT, a flavonoid found in fruits and vegetables, exhibits strong free radical-scavenging abilities and has been reported to protect against testicular toxicity induced by environmental pollutants^17^. Quercetin has emerged as a promising therapeutic agent for male infertility, primarily due to its potent antioxidant and anti-inflammatory properties. It effectively combats oxidative stress in the testes and spermatozoa by scavenging free radicals, reducing lipid peroxidation, and bolstering the endogenous antioxidant system, thereby preserving sperm quality, motility, membrane integrity, and mitochondrial activity^17,18^.

However, the conventional forms face limitations in bioavailability and targeted delivery, which can be overcome using nanotechnology-based formulations such as niosomes^19,20^. Nanotechnology is the management of material at the nanometer scale-one billion times smaller than a meter^21^. Targeted delivery appears to be a challenging but achievable task with the use of novel drug delivery systems such as nanoparticles^22,23^. Nanoparticles are seen as a promising technique for improving the efficiency of drugs such as tobramycin, cephalexin, and ciprofloxacin^24–26^. Therapeutic and pharmacological potential for improving a drug’s bioavailability, effectiveness, and selectivity was demonstrated by nanoparticles. Niosomes are a class of nanoparticles made up of bilayers of non-ionic surface-active substances that are often stabilized by the incorporation of cholesterol^27^. They are biodegradable, biocompatible, and chemically stable, have low toxicity, and improve targeting, bioavailability, and sustainability of drugs^27^.

The information about the effects of LC or QT, particularly the niosomal formulations, on male reproductive capacity, especially when exposed to environmental contaminants such as ATZ, is still scarce and therefore requires in-depth investigation. Additionally, the mechanism by which the two treatments exert their beneficial effects remains unclear. Therefore, the current study was conducted to evaluate the following: (1) elucidation of the beneficial roles of conventional LC and QT in mitigating ATZ toxicity in adult albino rats through evaluation of epididymal semen quality, serum male sex hormones and oxidative and anti-oxidative markers in testicular tissues. Furthermore, by evaluating their beneficial impacts on specific steroidogenic genes as well as histopathological assessment and lesion scoring of testicular, epididymal, seminal vesicular, and prostatic tissues, we intended to gain insight into the molecular mechanism by which LC and QT could alleviate ATZ toxicity in the testes. (2) Shedding light on the advantageous effects of LC and QT-loaded niosomes relative to their conventional counterparts in abating ATZ insults and elucidating the genetic pathways that contribute to the protection of testicular function from ATZ toxicity. By investigating these compounds in both conventional and niosomal formulations, this research will contribute to the development of more effective strategies to counteract ATZ-induced reproductive toxicity, ultimately improving male fertility outcomes.

## Materials and methods

### Materials

Span 60 and cholesterol were obtained from Agitech for Integrated Scientific Instruments Co., SAE, New Cairo City, Egypt. Chloroform, methanol and phosphate buffer were obtained from Cornell Lab for Life Science and Lab Equipment Co., Egypt.

### Drugs

#### L-carnitine

Chemical name of LC is (3R)-3-hydroxy-4-(trimethylazaniumyl)butanoate. Its generic name is L-carnitine with molecular formula of C7H15NO3 and molecular weight of 161.2 g/mol.

The supplier of LC crystals was Merck Co. (Darmstadt, Germany), with a purity of ≥ 98.0%. LC crystals were dissolved in distilled water to create a stock maintained in the refrigerator at 4 °C, and the stock was made every week^28^. The LC crystals were dissolved in distilled water to make a solution that achieved the recommended dose of 200 mg/kgb.wt.^29^.

### L-carnitine-loaded niosomes

#### Preparation of L-carnitine-loaded niosomes

L-carnitine-loaded niosomes (LCLN) formulation was prepared using thin film hydration method as described by^30^. Thirty mg (30 mg) of Span 60, cholesterol (molar ratio: 1:1), and 10 mg of LC were dissolved in a solution of methanol and chloroform in a round flask. The thin film was then created by rotary evaporating the entire mixture at 40 °C with decreased pressure. After drying, the thin layer was hydrated for two hrs. at 60 °C using 10 ml of phosphate buffer (PB) with a pH of 7.4 (10 mg). To separate the entrapped and unentrapped drugs, the produced formulation was sonicated for 30 min before being centrifuged for one hour at 15,000 rpm and 4 °C in a cooling centrifuge. The gathered niosome pellets were re-suspended in the distilled water for treating animals and the other produced formulation was stored at 4 °C for additional use after.

### In vitro characterization of L-carnitine-loaded niosomes (Particle size, poly-dispersity index, size distribution, and zeta potential determination)

The niosome formulation that was prepared was evaluated for particle size, polydispersity index (PDI), and zeta potential utilizing the Malvern PCS4700 Instruments^31^. To generate an appropriate scattering intensity, a sample of LCLN formulation was diluted with de-ionized water before measurement. Three independent measurements were taken at the room temperature.

### Transmission electron microscopy

With using a transmission electron microscopy (TEM), the morphology and shape of the LCLN formulation were examined^31^. A drop of a 1% aqueous solution of phosphotungstic acid dye was used to stain the freshly made sample, which was then placed onto the surface of a carbon-coated copper grid for TEM visualization. Images were captured at the appropriate magnifications.

L-carnitine-loaded niosomal solution 10.53% in distilled water was prepared in stock and was kept at 4 °C. Fresh solutions were prepared every 2 weeks, given by oral gavage of rats (stomach tube), at a dose of 0.19 ml (200 mg)/kg Rat^19^.

### Quercetin dihydrate

Chemical name of QT is 2-(3, 4-dihydroxyphenyl)-3,5,7-trihydroxychromen-4-one; dihydrate. The generic name is quercetin dihydrate with molecular formula of C_15_H_10_O_7_ and molecular weight of 338.27 g/mol.

Quercetin dihydrate powder was purchased from S. D. Fine Chem Ltd. Company, purity of ≥ 95% (HPLC), Mumbai, India. It is water-suspended, and the suspension was prepared by suspending it in sterile distilled water to make a stock every week^28^. QT suspension was made to meet the required dose in the current study of 25 mg/kg body weight^32^.

### Quercetin-loaded niosomes

#### Preparation of Quercetin-loaded niosomes formulations

Using the thin film hydration method, various Quercetin-loaded niosomes (QTLN) were formulated^33^. The calculated amounts of span 60, QT (10 mg), and cholesterol were dissolved in a solution (10 ml) of chloroform and methanol (2:1). After that, a Stuart rotary evaporator (RE300, UK) was used to evaporate this solution in a round-bottom flask at 100 rpm and 40 °C under vacuum. The resultant film was treated with 10 milliliters of PB at 60 degrees Celsius for two hrs. An ultrasonicator (Sonix, IL, USA) was used to sonicate QTLN formulations for 30 min. QTLN pellets were separated using a centrifuge (SIGMA, Germany) set to 15,000 rpm for an hour. QTLN pellets were stored at 4 °C after being diluted with 10 milliliters of distilled water.

#### In vitro characterization of quercetin-loaded niosomes (Particle Size, Poly-dispersity Index, size distribution, and Zeta Potential Determination)

Particle size and PDI are significant noisome characteristics that influence the particle’s dispersion, homogeneity, distribution, and subsequently targetability^34^. The electrostatic charge of the QTLN formulation was measured using the zeta potential in order to assess its stability and surface characteristics^35^. Using distilled water (9 ml), three samples of each QTLN formulation (1 ml) were diluted to assess the particle size and PDI in three replicates using dynamic light scattering (DLS, Malvern, Germany).

#### Transmission electron microscopy

A Transmission Electron Microscope (Carl Zeiss, Germany) was employed for detection of the shape and surface properties of QTLN vesicles as they formed^36^. With a carbon-coated copper grid, a 20 µL sample of the optimum QTLN formulation has been used and dyed with phosphotungstic dye before it was dried. The image was examined at different magnifications using TEM.

Quercetin-loaded niosomal solution 2.5% in distilled water was prepared in a stock for oral gavage by stomach tube, and was kept at 4 °C. Fresh solutions were prepared every 2 weeks, given at a dose of 0.1 ml (25 mg)/kg b.wt. Rat^37^.

#### Atrazine

Chemical name of ATZ is 6-chloro-4-*N*-ethyl-2-*N*-propan-2-yl-1,3,5-triazine-2,4-diamine. The Generic name is Gesaprim with molecular formula of C_8_H_14_ClN_5_ and molecular weight of 215.68 g/mol.

The toxicity experiments were conducted using ATZ (organo-chlorine herbicide) with 80% purity. ATZ wettable powder (80% W/W) was bought from Syngenta Company and imported by El-Nasr Pharmaceutical Co. in Egypt.

Atrazine powder was suspended in sterile distilled water to produce a suspension that achieved the recommended 120 mg/kg. b.wt./dose. The suspension was freshly prepared every day^38^. The selected dose of 120 mg/kg body weight is a well-established one for inducing significant and consistent testicular oxidative stress and spermatotoxicity in rodent models, as extensively documented in the literature^39^. This dosage is recognized for its ability to reliably decrease sperm quality and testosterone levels, and to induce histopathological changes in testicular tissue, making it a suitable positive control for evaluating the protective effects of potential therapeutic agents^40^. While this dose is considerably higher than typical environmental residues (found in the range of micrograms per kilogram), it is intentionally used in high-dose toxicological models to elucidate mechanisms of toxicity and assess mitigation strategies under controlled conditions^41^.

#### Animals

A total of 30 adult male Wistar rats (average weight of 140–150 g b.wt.) were obtained from the lab animal facility in Helwan, Egypt, for this investigation. All animals were housed in plastic cages and fed a conventional rodent diet (21% protein, 3% crude fiber, 7% fat, 1% vitamins, and mineral premix, and 68% yellow maize, or “2490.00 Kcal/kg”) on a daily basis. Potable water from readily accessible sources was available around the clock. The experiment was conducted using a conventional 12-hour light and dark procedure at a temperature of 22 ± 2 °C.

The experimental methods were started following a 15-day acclimatization period. To determine the appropriate dosages for each therapy, the weights of animals were recorded weekly.

### Ethical statement

The experimental design and reporting followed the ARRIVE guidelines to ensure transparency and reproducibility. Based on these principles, a detailed experimental protocol was prepared and submitted for ethical review. In order to ensure complete compliance with both institutional and international standards for animal research, all following operations, such as animal housing and care, dosing regimen, blood collection, and euthanasia, were carried out strictly in line with this authorized protocol.

The work on animals in this study was carried out in accordance with the rules of the Institutional Review Board of the Ethical Committee of Animal Care and Use in Scientific Research of the Faculty of Veterinary Medicine, Minia University.

### Approval statement

This study was approved by the Institutional Review Board of the Ethical Committee for Animal Care and Use in Scientific Research, Faculty of Veterinary Medicine, Minia University (IRB-FVM-MU) under approval number IRB-FVM-MU-2024-108.

### Experimental design

Following acclimation, the animals were divided equally into six groups of five rats each.

1) Group I (Control; *n* = 5): Rats in this group were given sterile distilled water.

2) Group II (ATZ group; *n* = 5): The rats in this group were given a daily dose of 120 mg/kg. b.wt. ATZ solution^39^.

3) Group III (ATZ + LC; *n* = 5): Rats in this group were given a daily dose of 200 mg/kg. b.wt. LC solution^29,40^, one hour before being gavaged with 120 mg/kg. b.wt. ATZ solution.

4) Group IV (ATZ+LCLN; *n* = 5): Rats in this group were given a daily dosage of 200 mg/kg. b.wt. LCLN^19^ one hour before gavaging with 120 mg/kg. b.wt. ATZ solution.

5) Group V (ATZ + QT; *n* = 5): The rats in this group were given a daily dose of 25 mg/kg. b.wt. QT^32^ one hour before being gavaged with 120 mg/kg. b.wt. ATZ solution.

6) Group VI (ATZ+QTLN; *n* = 5): Rats in this group were given a daily dosage of 25 mg/kg. b.wt. QTLN solution^37^ one hour before being gavaged with 120 mg/kg. b .wt. ATZ solution.

All treatments, including the control, were given using stomach tube gavage once daily for consecutive 56 days (8 weeks) to cover one complete spermatogenic cycle of the rat.

### Relative and absolute organ weights

Absolute organ weights were reported by weighing the right testis, left testis, epididymis, seminal vesicles and prostate using a digital balance. Furthermore, the relative organ weights (g/g %) for all these organs were also recorded by the following equation^41^.

$${\mathrm{Relative~organ~weight~}}\left( {\frac{{\mathrm{g}}}{{\mathrm{g}}}{{\% }}} \right) = \frac{{weight~of~the~organ~\left( g \right)}}{{final~body~weight~\left( g \right)}}~x100$$

### Collection of blood samples

At the end of the study, each rat was anesthetized using Ketamine HCL 90 mg/kg. b.wt. and Xylazine HCL 5 mg/kg. b.wt. according to Dinca et al.^42^, then samples of blood were collected from each rat individually, from the retro-orbital venous plexus. Blood sample was collected in serum gel tubes and then allowed to coagulate at room temperature before being centrifuged at 3000 rpm for 15 min. The clear non-hemolyzed supernatant serum was rapidly aspirated and maintained at -20° C for further analysis of steroid sex hormones (Testosterone, FSH and LH) and serum levels of oxidative /antioxidative stress markers (MDA, catalase “CAT”, superoxide dismutase “SOD” and glutathione peroxidase “GPx”).

### Serum biochemical analysis

#### Blood levels of testosterone, FSH and LH hormones

Specific ELISA kits were used, and according to the instruction of the manufacturer, serum concentration of testosterone, FSH and LH hormones were estimated. Concentration of testosterone was measured using Rat Testosterone ELISA Kit obtained from CAUSABIO Company, Houston, TX 77,054, USA, and Catalogue No.: CSB-E05100r. For FSH, its levels were measured using Rat FSH ELISA Kit, Catalogue No.: ER0960, Wuhan Fine Biotech Co., Ltd., Wuhan, Hubei, China. LH levels in blood were measured using Rat LH ELISA Kit, Catalogue No.: CSB-E12654r, CAUSABIO Company, Houston, TX 77,054, USA.

### Serum malondialdehyde, catalase, superoxide dismutase and glutathione peroxidase

Concerning MDA and CAT, their serum levels were estimated using particular colorimetric assay kits (Biodignostic Company, Giza, Egypt; Catalogue numbers: MD 25 29 and CA 25 17 for MDA and CAT, respectively). In addition, SOD was measured using specific colorimetric assay kits (Arbor Assays Company, 1514 Eisenhower Place Ann Arbor, Michigan 48108 USA, Catalog Number K028-H1), while GPx was measured using particular colorimetric assay kits (BioAssay Systems Company, Hayward, CA 94545, USA, Catalog Number: EGPX-100) following the manufacturer’s instructions for each test.

### Epididymal semen collection and evaluation

At the end of the investigation, following blood collection, the rats were euthanized by cervical dislocation, and the left epididymis was retrieved instantly after separation and homogenized in sterile saline by maceration of the epididymis’ tail with a sterile scalpel. Computer-Assisted Semen Analysis, or CASA (image analysis with a phase-contrast microscope and computer assessments of motion parameters), objectively examined sperm motility characteristics, morphology, and concentration, as detailed by Slott et al.^43^. The CASA system (SCA^®^, Sperm Class Analyzer, Microptics, Spain) consists of a high-resolution camera (Basler acA1300-200uc, Basler, Germany) connected to a phase-contrast microscope (Nikon Eclipse E200, Nikon) using a 10× objective (Nikon 10×/0.25 Ph1 BM, Nikon). The CASA system was used to analyze several images among the different groups (supplementary file, Fig.S4-Fig.S9).

### Analysis of mRNA relative expression levels of some genes using Real-Time PCR System

Specimens from testes were collected after blood sampling and sacrifice, in Eppendorf’s tubes and kept at -20° C for genetic examination.

Tissue samples were subjected to total RNA isolation with the RNeasy Mini Kit (Qiagen Cat. No./ID 74104). SuperScript Reverse Transcriptase (Thermoscientific) was used to synthesize first-strand cDNA following the manufacturer’s instructions^44^. Following the manufacturer’s instructions, quantitative PCR was carried out using an ABI Prism StepOnePlus Real-Time PCR System (Applied Biosystems) and PowerTrackTM SYBR Green Master Mix Applied BiosystemsTM^45,46^. The evaluated genes’ primer sets were gathered and are shown in Table 1. After normalizing the target mRNA expression to *ACTB*, the normalized CT method was used to calculate the fold change in gene expression^47,48^.

**Table 1:** The primer sequences of the target genes.

Primer	Sequence	Amplicon size (bp)	Accession number
*StAR*	F: TGGCTGCCAAAGACCATCAT R: TGGTGGGCAGTCCTTAACAC	241	NM_031558.3
*CYP11A1*	F: GCAAAAGGTCTTTGCCTGCG R: TGGATTCTGTGTGTGCCGTT	212	NM_017286.3
*Hsd-3b*	F: CTCACATGTCCTACCCAGGC R: ATTTTTGAGGGCCGCAAGT	362	NM_001007719.3
*ACTB*	F: CGCGAGTACAACCTTCTTG R: AGTTGGTGACAATGCCGTG	297	NM_031144.3

### Organ collection for histopathological examination

By the end of the experiment, following sacrificing of rats, specimens from the testes, prostate gland, vesicular glands and epididymis were dissected carefully then washed several times using physiological saline (NaCl 0.9%), before a piece of these organs was collected and fixed in neutral buffered formalin 10% solution. The specimens were dehydrated with increasing grades of ethyl alcohol, then cleaned in xylene, soaked in soft paraffin, and lastly embedded in hard paraffin. Sections of 4–6 μm were cut with a rotatory microtome and put on clean, dry glass slides. The collected slides were stained with Hematoxylin and Eosin (H&E) for histological inspection using a LEICA (DFC290 HD system digital camera, Heerbrugg, Switzerland) attached to a light microscope with 10, 20, and 40 objective lenses^49^.

In addition, 5 random fields/sections/organs were examined and scored semiquantitatively. A table was created to show the kind, frequency, and severity of histopathological lesions in each group^50^.

**Fig. 1:**
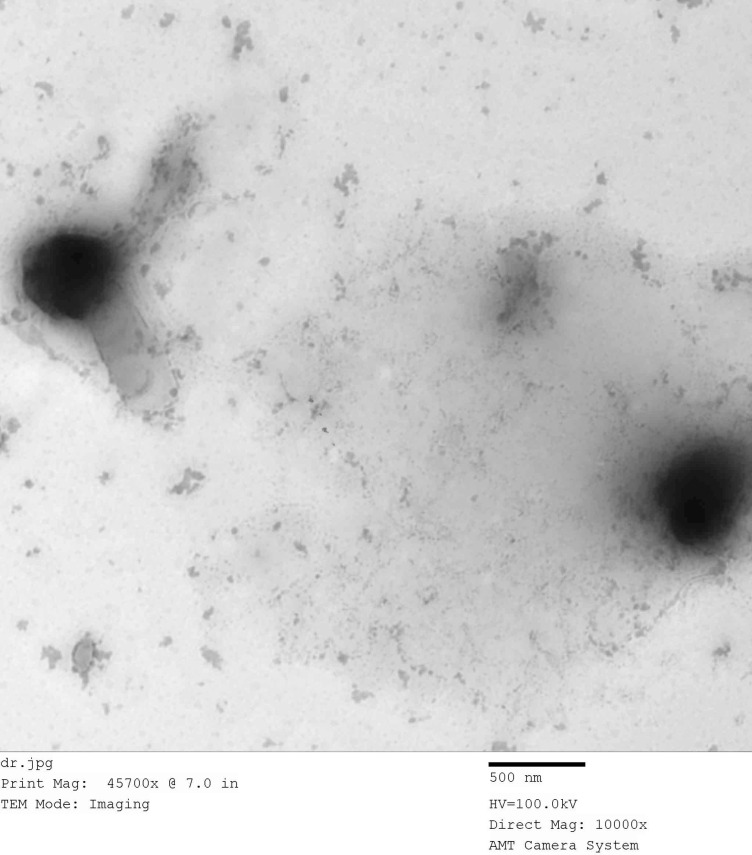
Surface morphology of L-carnitine-loaded niosomes formulation by TEM.

**Fig. 2:**
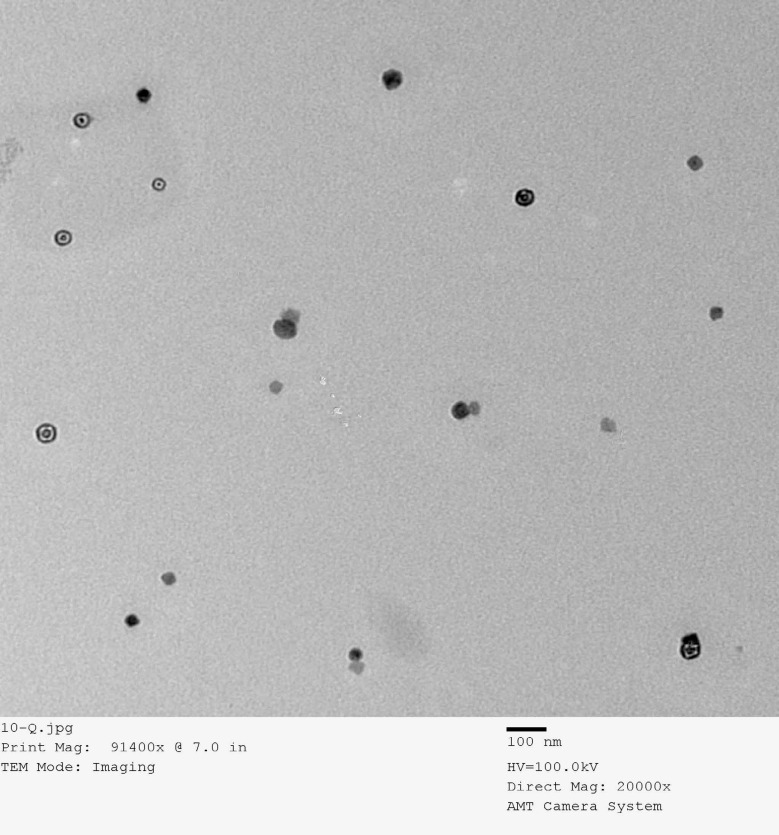
TEM image of optimum quercetin-loaded niosomes formulation.

**Table 2:** Absolute organ weights in control and treated animals (Mean ± SEM) (*n* = 5).

sex organs absolute weight (g)	Control	ATZ	ATZ + LC	ATZ+LCLN	ATZ + QT	ATZ+QTLN
Right testis	1.52 ± 0.04	1.59 ± 0.08	1.36 ± 0.17	1.50 ± 0.08	1.50 ± 0.06	1.47 ± 0.05
Left testis	1.54 ± 0.06	1.60 ± 0.08	1.50 ± 0.06	1.62 ± 0.17	1.52 ± 0.06	1.49 ± 0.05
Epididymis	0.52 ± 0.03	0.42 ± 0.04	0.42 ± 0.05	0.41 ± 0.02	0.41 ± 0.01	0.43 ± 0.03
Seminal vesicle	1.82 ± 0.18	0.94 ± 0.09^a^	1.10 ± 0.10^a^	1.20 ± 0.12^a^	1.32 ± 0.07^a^	1.20 ± 0.06^a^
Prostate	0.51 ± 0.06	0.39 ± 0.03	0.38 ± 0.06	0.38 ± 0.03	0.42 ± 0.04	0.45 ± 0.06

SEM: Standard error of the mean, ATZ: Atrazine, LC: L-carnitine, LCLN: L-carnitine-loaded niosomes, QT: Quercetin, QTLN: Quercetin-loaded niosomes. Data are expressed as mean ± standard error of the mean (*n* = 5). ^a^ *P* < 0.001 versus the control group.

**Table 3:** Relative organ weights in control and treated animals (Mean ± SEM) (*n* = 5).

Organ relative weight g/100 g b wt.	Control	ATZ	ATZ + LC	ATZ+LCLN	ATZ + QT	ATZ+QTLN
Right Testis	0.64 ± 0.04	0.73 ± 0.031	0.67 ± 0.059	0.72 ± 0.032	0.70 ± 0.035	0.68 ± 0.027
Left Testis	0.65 ± 0.043	0.74 ± 0.038	0.76 ± 0.030	0.77 ± 0.070	0.71 ± 0.034	0.69 ± 0.024
Epididymis	0.22 ± 0.017	0.19 ± 0.016	0.21 ± 0.017	0.20 ± 0.007	0.19 ± 0.007	0.20 ± 0.012
Seminal Vesicle	0.76 ± 0.074	0.44 ± 0.038^a^	0.56 ± 0.060	0.57 ± 0.048	0.61 ± 0.044	0.56 ± 0.016
Prostate	0.21 ± 0.019	0.18 ± 0.015	0.19 ± 0.031	0.18 ± 0.017	0.20 ± 0.020	0.21 ± 0.023

SEM: Standard error of the mean, ATZ: Atrazine, LC: L-carnitine, LCLN: L-carnitine-loaded niosomes, QT: Quercetin, QTLN: Quercetin-loaded niosomes.

Data are expressed as mean ± standard error of the mean (*n* = 5). ^a^ *P* < 0.001 versus the control group.

**Fig. 3:**
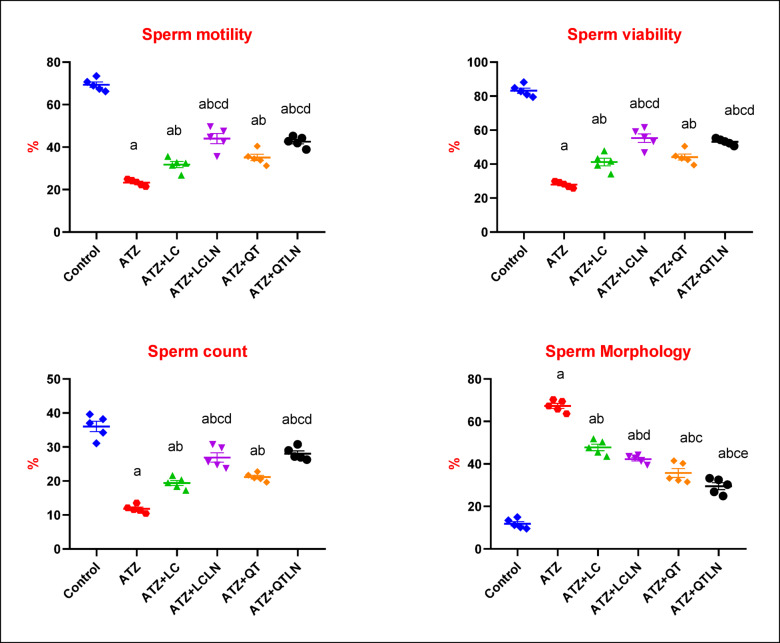
Semen characteristics in control and treated animals. ATZ: Atrazine, LC: L-Carnitine, LCLN: L-carnitine-loaded niosomes, QT: Quercetin, QTLN: Quercetin-loaded niosomes. Data are expressed as mean ± standard error of the mean. ^a^*P* < 0.001 versus control group(*n* = 5), ^b^*P* < 0.01 versus ATZ group, ^c^*P* < 0.001 versus ATZ + LC group, ^d^*P* < 0.01 versus ATZ + QT group, ^e^*P* < 0.001 versus LCLN group.

**Table 4:** Serum sex hormones and oxidative stress markers in control and treated animals.

Group	Control	ATZ	ATZ + LC	ATZ+LCLN	ATZ + QT	ATZ+QTLN
Testosterone (ng/ml)	3.99 ± 0.28	0.56 ± 0.05^a^	1.77 ± 0.06^ab^	2.22 ± 0.07^abd^	1.50 ± 0.08^ab^	1.83 ± 0.07^ab^
FSH (mIU/ml)	11.50 ± 0.81	1.64 ± 0.14^a^	5.12 ± 0.17^ab^	6.44 ± 0.19^abd^	4.49 ± 0.22^ab^	4.93 ± 0.35 ^ab^
LH (mIU/ml)	6.39 ± 0.45	0.91 ± 0.08 ^a^	2.84 ± 0.09 ^ab^	3.57 ± 0.11 ^abd^	2.48 ± 0.13 ^ab^	2.74 ± 0.17 ^ab^
MDA (nmol/ml)	0.96 ± 0.05	3.83 ± 0.12 ^a^	2.76 ± 0.08 ^ab^	2.27 ± 0.03 ^abcd^	2.71 ± 0.05 ^ab^	2.23 ± 0.03 ^abcd^
SOD (U/ml)	2.88 ± 0.05	0.84 ± 0.06 ^a^	1.61 ± 0.08 ^ab^	2.11 ± 0.06 ^abcd^	1.62 ± 0.08 ^ab^	2.13 ± 0.04 ^abcd^
GPx (U/L)	87.62 ± 2.10	44.24 ± 1.09 ^a^	53.06 ± 1.21 ^ab^	67.40 ± 2.03 ^abc^	59.99 ± 1.08 ^ab^	68.22 ± 3.30 ^abc^
CAT (U/L)	3.89 ± 0.09	1.06 ± 0.04 ^a^	2.38 ± 0.03 ^ab^	2.67 ± 0.04 ^abcd^	2.42 ± 0.04 ^ab^	2.83 ± 0.05 ^abcd^

ATZ: Atrazine, LC: L-carnitine, LCLN: L-carnitine-loaded niosomes, QT: Quercetin, QTLN: Quercetin-loaded niosomes, FSH: Follicle stimulating hormone, LH: Luteinizing hormone, MDA: Malondialdehyde, SOD: Superoxide dismutase, GPx: Glutathione peroxidase, CAT: Catalase.

Data are expressed as mean ± standard error of the mean (n=5).

In the same row,^a^

P< 0.001 versus control group, ^b^

P< 0.01 versus ATZ group, ^c^

P< 0.001 versus ATZ+LC group, ^d^

P< 0.01 versus ATZ+QT group.

**Fig. 4:**
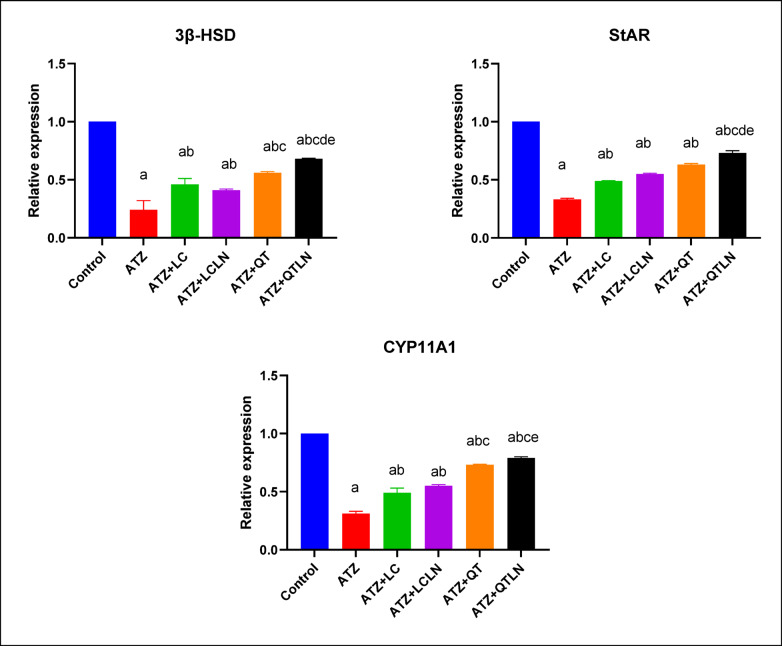
Steroidogenic gene analysis in the testis of control and treated animals (Mean ± S.E.). ATZ: Atrazine, LC: L-Carnitine, LCLN: L-carnitine-loaded niosomes, QT: Quercetin, QTLN: Quercetin-loaded niosomes, *3β-HSD*: 3β-Hydroxysteroid dehydrogenase, *StAR*: steroidogenic acute regulatory protein, *CYP11A1*: Cytochrome P450 Family 11 Subfamily A Member 1. Data are expressed as mean ± standard error of the mean (*n* = 5). ^a^*P* < 0.05 versus control group, ^b^*P* < 0.05 versus ATZ group, ^c^*P* < 0.05 versus ATZ + LC group, ^d^*P* < 0.05 versus ATZ + QT group, ^e^*P* < 0.05 versus LCLN group.

**Fig. 5:**
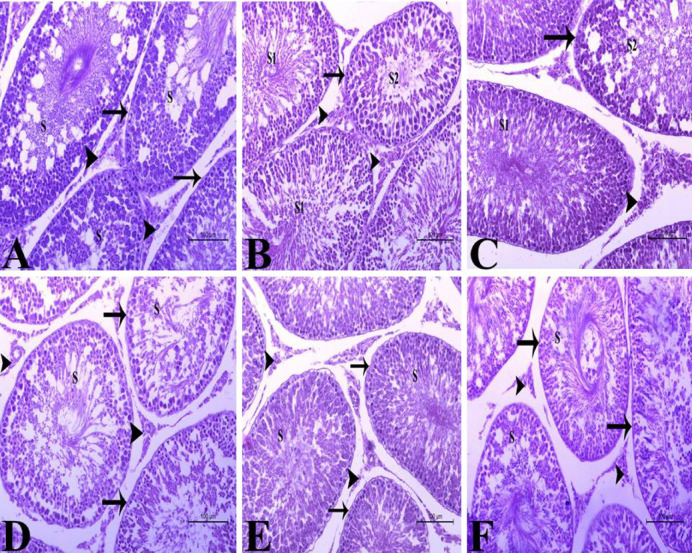
A histopathological picture of testicular tissues in adult male albino rats of the studied groups showing: **A**: control group showed normal picture of testicular tissue including normal seminiferous tubules (S) enclosed by thin basal lamina (arrow), lined with normal spermatogenic cells and contained huge amount of spermatozoa. The Leydig cells (arrowhead) appeared active. **B**: Atrazine-treated group showed seminiferous tubules (S2) enclosed by very thick basal lamina (arrow), lined with degenerated spermatogenic cells, few spermatid and sperms while seminiferous tubules (S1) lined with normal spermatogenic cells with few spermatid and spermatozoa. Note, the interstitial tissue showed marked inactive Leydig cells (arrowhead). **C**: Atrazine + L-carnitine group showed seminiferous tubules (S1) enclosed by thick basal lamina (arrow), lined with degenerated spermatogenic cells, few spermatid and sperms while seminiferous tubules (S2) appeared normal. The interstitial Leydig cells (arrowhead) became active. **D**: Atrazine + L-carnitine-loaded niosomes group exhibited normal seminiferous tubules (S) enclosed by thin basal lamina (arrow), lined with normal spermatogenic cells and containing spermatozoa. The Leydig cells (arrowhead) appeared active. **E**: Atrazine + quercetin-treated group showed normal seminiferous tubules (S) enclosed by thin basal lamina (arrow), lined with normal spermatogenic cells and contained spermatozoa. The Leydig cells (arrowhead) appeared active. **F**: Atrazine + quercetin-loaded niosomes-treated group showed normal seminiferous tubules (S) enclosed by thin basal lamina (arrow), containing huge amount of spermatozoa. The Leydig cells (arrowhead) appeared active. (H&E stain X200).

**Fig. 6:**
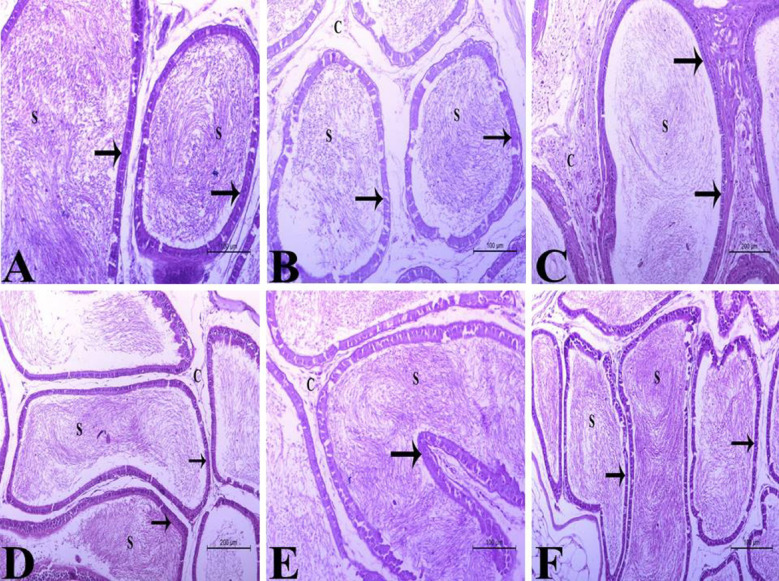
A histopathological picture of epididymis in studied groups showing: **A**: control group showed normal histological orientation of the epididymal tubule separated by very scant amount of connective tissue (C) and lined with normal epithelium (arrow) containing huge amount of sperms (S). **B**: Atrazine-treated group showed the epididymal tubule separated by very thick connective tissue (C) and lined with degenerated epithelium (arrow) containing little amount of sperms (S). **C**: Atrazine + L-carnitine-treated group exhibited the epididymal tubule separated by moderate amount of connective tissue (C) and lined with normal epithelium (arrow) containing considerable amount of sperms (S1) while other tubules contained few amount of sperms (S2). **D**: Atrazine + L-carnitine-loaded niosomes appeared normal epididymal tubule separated by very scant amount of connective tissue (C) and lined with normal epithelium (arrow) containing huge amount of sperms (S). **E**: Atrazine + quercetin-treated group showed normal epididymal tubules separated by very scant amount of connective tissue (C) and lined with normal epithelium (arrow) containing large amount of sperms (S). **F**: Atrazine + quercetin-loaded niosomes-treated group showed normal epididymal tubules separated by very scant amount of connective tissue (C) and lined with normal epithelium (arrow) containing huge amount of sperms (H&E stain X200).

**Fig. 7:**
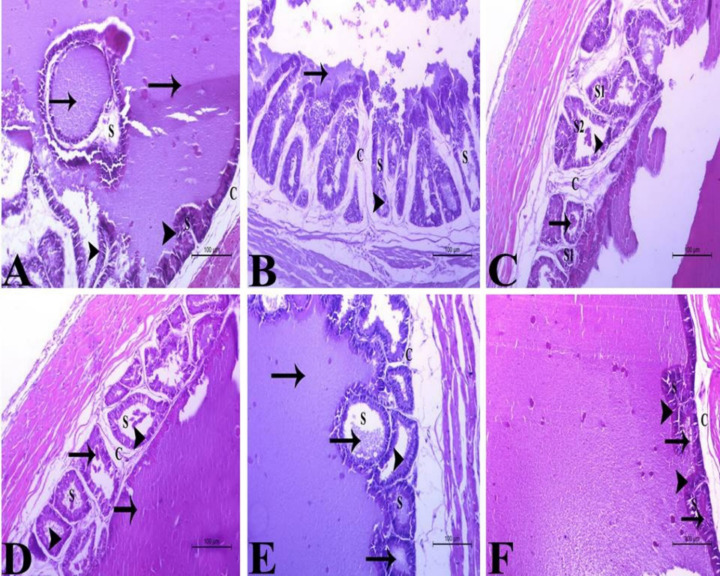
A histopathological picture of seminal vesicular gland in adult male rats of the studied groups showing: **A**: control group showed normal architecture of the vesicular gland with secretory acini (S) lined with high columnar secretory epithelium (arrowhead). The lumen of secretory acini contained massive amount of secretion (arrow). The-inter acinar connective tissue (C) was very scant. **B**: Atrazine-treated group showed collapsed and inactive acini (S) lined with inactive epithelium, the secretory materials were very scant. The inter-acinar connective tissue (C) was very thick. **C**: Atrazine + L-carnitine-treated group showed collapsed secretory acini (S1) while other secretory acini (S2) were lined with active secretory epithelium (arrowhead). The lumen of secretory acini contained little amount of secretion (arrow). The-inter acinar connective tissue (C) was moderate. **D**: Atrazine + L-carnitine-loaded niosomes-treated group showed secretory acini (S) lined with high columnar secretory epithelium (arrowhead). The lumen of secretory acini contained massive amount of secretion (arrow). The-inter acinar connective tissue (C) was very scant. **E**: Atrazine + quercetin- treated group showed secretory acini (S) lined with high columnar secretory epithelium (arrowhead). The lumen of secretory acini contained massive amount of secretion (arrow). The-inter acinar connective tissue (C) was very scant.**F**: Atrazine + quercetin-loaded niosomes-treated group showed secretory acini (S) lined with high columnar secretory epithelium (arrowhead). The lumen of secretory acini contained massive amount of secretion (arrow). The-inter acinar connective tissue (C) was very scant. (H&E stain X200).

**Fig. 8:**
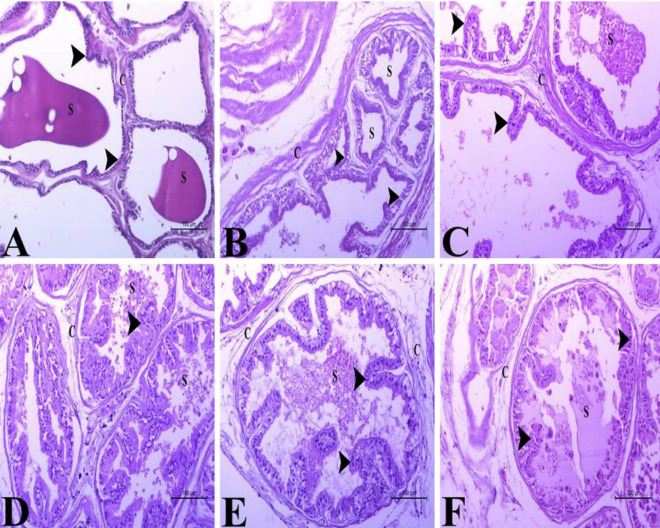
A histopathological picture of prostatic tissue in adult male albino rats of the studied groups showing: **A**: control group showed normal orientation of the prostatic tissue in which the acini separated by few amount of connective tissue (C) and lined with high columnar secretory epithelium (arrowhead). The lumen of secretory acini contained prostatic secretion (S). **B**: Atrazine-treated group showed small and inactive acini (S) separated by thick connective tissue (C). The acini were lined by inactive cuboidal epithelium with low secretory activity (arrowhead). The secretory materials disappeared from the acinar lumen. **C**: Atrazine + L-carnitine-treated group appeared with few normal acini separated by few amount of fibrous tissue (C) and lined with high columnar secretory epithelium (arrowhead). The lumen of secretory acini contained few secretory materials (S). **D**: Atrazine + L-carnitine-loaded niosomes-treated group displayed normal orientation of the prostatic tissue in which the acini were separated by few amount of connective tissue (C) and were lined with high columnar secretory epithelium (arrowhead). The lumen of secretory acini contained prostatic secretion (S). **E**: Atrazine + quercetin-treated group showed normal orientation of the prostatic tissue in which the acini were separated by few amount of connective tissue (C) and were lined with high columnar secretory epithelium (arrowhead). The lumen of secretory acini contained prostatic secretion (S). **F**: Atrazine + quercetin-loaded niosomes-treated group showed normal orientation of the prostatic tissue in which the acini were separated by few amount of connective tissue (C) and were lined with high columnar secretory epithelium (arrowhead). The lumen of secretory acini contained prostatic secretion (S). (H&E stain X200).

**Table 5:** Histopathological scoring of the sex organs’ tissue injury.

Group	Testis			Prostate gland				Vesicular gland				Epididymal sperm content
	Degeneration of spermatogenic cells	Depletion of sperms	Congestion of testicular vessels	Fibrosis	Secretory acini collapsing	Activity of secretory epithelium	Secretory materials	Fibrosis	Secretory acini collapsing	Activity of secretory epithelium	Secretory materials	
Control	0	0	0	0	0	4	4	0	0	4	4	4
ATZ	2	3	3	3	3	1	1	2	3	1	1	1
ATZ + LC	0	1	1	1	1	3	3	1	1	3	3	2
ATZ+LCLN	0	0	0	0	0	4	4	0	0	4	4	4
ATZ + QT	0	0	0	0	0	4	4	0	0	4	4	4
ATZ+QTLN	0	0	0	0	0	4	4	0	0	4	4	4

Histopathological scoring of sex organs’ tissue injury was scaled in degrees as follows: 0 = no change; 1 ≤ 25% tissue damage; 2 = 26–50% tissue damage; 3 = 51–75% tissue damage; 4 = 76–100% tissue damage

ATZ: Atrazine, LC: L-Carnitine, LCLN: L-carnitine-loaded niosomes, QT: Quercetin, QTLN: Quercetin-loaded niosomes.

### Statistical analysis

Results were presented as mean ± SEM. Statistical significance was established using one-way analysis of variance (ANOVA) and Tukey’s post-hoc test for multiple comparisons in SPSS (version 20.0) software (IBM SPSS Statistic 20.0, Armonk, NY, USA). *P* values < 0.05 were regarded as statistically significant.

## Results

### Characterization of L-carnitine-loaded niosomes

The electron transmission microphotograph of LCLN formulation was shown in Fig. 1 and Fig. S2. TEM analysis is a useful method for asserting the results of particle size analysis and examining the morphology of colloidal systems. This figure shows that the nanoparticles are approximately spherical with smooth surface, appearing as black dots, well dispersed without aggregation on the surface.

### In vitro characterization of L-carnitine-loaded Niosomes (Particle Size, Size Distribution, and Zeta potential)

Because smaller vesicles have a larger surface area and greater drug absorption, vesicle size is crucial for assessing the effectiveness of niosomes. The LCLN formulation showed a small particle size of 241.7 ± 1.5 nm, suggesting formation of nano-sized vesicles. The PDI was used to quantify the extent of dispersion, distribution of size, and vesicle size uniformity within a sample. The PDI analysis of the prepared formulation revealed a low PDI of 0.472, which supported the presence of homogeneity and a narrow distribution of particle size. Zeta potential, which gauges the charges that the vesicles have acquired, can be used to evaluate the stability of colloidal dispersions. The prepared formulation possessed a negative surface charge of -37.7 ± 0.4 mV, indicating good physical and chemical stability, enough for electrostatic stabilization.

### Characterization of quercetin-loaded niosomes

Figure 2 and Fig. S3 depicted the optimal QTLN formulation’s shape. The vesicles exhibited spherical vesicular structures with a well-defined outline and core, appearing as black dots without aggregation on the surface.

### In Vitro characterization of optimum QTLN formulation (Particle Size, Polydispersity Index, and Zeta Potential)

The optimum QTLN formulation showed a small particle size of 291.1 ± 5.2 nm with a low PDI of 0.471, indicating a consistent size distribution, small interfacial tension, a homogeneous niosome, and fewer tendencies for aggregation.

The optimal QTLN formulation’s zeta potential was determined to be − 26.7 ± 2.20 mV. The surface possessed a negative charge, which is beneficial for electrostatic stabilization, according to the zeta potential value. The QTLN formulation’s negative charge created electrostatic repulsions between vesicles, resulting in stable vesicles and increased vesicle permeability.

### Reproductive organs weight in control and treated animals

The absolute and relative weights of the testes, epididymis, seminal vesicles, and prostate are presented in Tables 2 and 3. The mean values of relative and absolute weights of right and left testis, epididymis, and prostate gland did not differ significantly among all groups (*P* > 0.05). However, ATZ remarkably decreased the relative and absolute seminal vesicle weights compared to control groups (*P* < 0.001). On the other side, when animals were co-administered ATZ with the different treatments, the decreased relative and absolute seminal vesicle weights recovered slightly, but not significantly (*P* > 0.05).

### Epididymal semen characteristics in control and treated animals

The results of sperm cell characteristics are demonstrated in Fig. 3. Compared to the control group, ATZ significantly reduced sperm cell concentration (3.06-fold, Table S1), as well as percentages of sperm cell motility (2.97-fold) and viability (2.97-fold) while increasing the percentages of sperm cell abnormalities (5.67-fold) (*P* < 0.001). On the other side, co-administration of either LC, LCLN, QT or QTLN with ATZ dramatically improved sperm quality by raising sperm cell concentration (1.65, 2.28, 1.79 and 2.38-fold, respectively, Table S2), motility (1.36, 1.89, 1.51 and 1.83-fold, respectively) and viability (1.47, 1.97, 1.58 and 1.90-fold, respectively) along with reducing sperm abnormalities (1.41, 1.59, 1.88 and 2.28-fold, respectively) when compared to the ATZ group (*P* < 0.01).

Furthermore, the improving activity for LCLN (*P* < 0.001) and QTLN (*P* < 0.01) was observed to be greater than their corresponding conventional form in terms of their effect on sperm cell motility (1.89 vs.1.36-fold and 1.83 vs. 1.51-fold, respectively, Table S2), viability (1.97 vs. 1.47-fold and 1.90 vs. 1.58-fold, respectively) and concentration (2.28 vs. 1.65-fold and 2.38 vs. 1.79-fold). However, the effects of LCLN and QTLN on sperm morphology were found to be equivalent to their conventional form (*P* > 0.05); at the same time, the enhancing effects of QT were superior to LC (*P* < 0.01), and the effect of QTLN was superior to LCLN (*P* < 0.001) in improving sperm morphology.

### Hormonal assay in control and treated animals

As shown in Table 4, serum levels of testosterone (7.13-fold, Table S1), LH (7.01-fold) and FSH (7.02-fold) were significantly reduced after 56 days of exposure to ATZ, when compared to the control group (*P* < 0.001). On the other hand, the deleterious effects of ATZ on such hormones were seen to be significantly ameliorated when rats were co-administered LC, LCLN, QT, or QTLN with ATZ (*P* < 0.01) (3.16, 3.96, 2.68 and 3.27-fold, respectively for testosterone; 3.12, 3.93, 2.74 and 3.01-fold, respectively for FSH; 3.12, 3.92, 2.73 and 3.01-fold, respectively for LH, Table S2).

Furthermore, LCLN and QTLN increased blood testosterone, LH, and FSH levels more than their conventional counterparts, albeit not significantly (*P* > 0.05) (Table 4).

### Serum oxidative stress markers in control and treated animals

The influence of ATZ as well as ATZ and co-treatment with QT and LC in niosomal and conventional forms on serum oxidant-antioxidant markers was shown in Table 4. The levels of MDA, SOD, GPx and CAT were markedly impaired in rats administered with ATZ alone when compared to control group, with a significant increase in MDA activity (3.99-fold, Table S1) and a substantial reduction in SOD (3.43-fold), GPx (1.98-fold) and CAT (3.67-fold) activities (*P* < 0.001).

However, co-treatment with QT and LC, either the niosomal or conventional form, resulted in an impressive amelioration in oxidant/antioxidant indicators, with decreased MDA levels (1.39, 1.69, 1.41 and 1.72-fold, respectively, Table S2) and increased SOD (1.92, 2.51, 1.93 and 2.54-fold, respectively), GPx (1.20, 1.52, 1.36 and 1.54-fold, respectively) and CAT levels (2.25, 2.52, 2.28, 2.67-fold, respectively) compared to ATZ group (*P* < 0.01).

However, it was found that QTLN (*P* < 0.01) and LCLN (*P* < 0.001) had a higher potential effect to mitigate ATZ-induced oxidative stress than their respective conventional forms, with the exception of GPx, which demonstrated a non-significantly higher increase with QTLN than QT but a significantly higher increase with LCLN than LC.

### The mRNA relative expression levels of *3β-HSD*,* StAR and CYP11A1* in the testis of control and treated animals

As depicted in Fig. 4, ATZ significantly reduced the mRNA relative expression levels of *3β-HSD (*4.17-fold), *StAR* (3.03-fold), and *CYP11A1* (3.23-fold) in testes (*P* < 0.05) in relation to control group. The most remarkable discovery is that all treatments (LC, LCLN, QT, and QTLN) significantly (*P* < 0.05) upregulated the expression of these genes (1.92, 1.71, 2.33, 2.83-fold, respectively, for *3β-HSD;* 1.48, 1.67, 1.91, 2.21-fold, respectively for *StAR;* 1.58, 1.77, 2.35, 2.55-fold, respectively for *CYP11A1*, Table S2), thereby significantly (*P* < 0.05) reducing the deleterious effects of ATZ.

Furthermore, for LC, the niosomal form performed similarly to the conventional one in terms of their effect on mRNA relative testicular expression of all studied genes. On the other hand, QTLN outperformed QT in terms of the enhancement of *3β-HSD* and *StAR* expression (*P* < 0.05).

In addition, the act of QT was better than LC (*P* < 0.05) in terms of its improving effects on *3β-HSD* and *CYP11A1* testicular expression while QTLN was more effective (*P* < 0.05) than LCLN regarding its effects on all studied genes (*3β-HSD*,* StAR*,* and CYP11A1*).

### Histopathological findings in testicular, epididymal, semen vesicle glandular and prostatic structures in control and treated rats

The histological results of the various organs under investigation, such as testes, prostate glands, seminal vesicles and epididymis, were depicted in Figs. 5, 6, 7 and 8; Table 5. It was clear that ATZ induced degenerative histological changes in testicular (Fig. 5 B), epididymal (Fig. 6 B), seminal vesicular gland (Fig. 7 B) and prostatic tissues (Fig. 8 B). Interestingly, co-treatment with LC, LCLN, QT and QTLN significantly ameliorated all of these negative changes. Furthermore, the ameliorative effects were obvious in the niosomal groups compared to the conventional form.

In addition, higher magnification images (Supplementary file, Fig.S1) confirmed the degenerative changes triggered by ATZ in the testis including degenerated spermatogenic cells with inactive Leydig cells (Fig. S1B). On the contrary, treatment with LC (Fig.S1C), LCLN (Fig.S1D), QT (Fig.S1E) or QTLN (Fig.S1F) significantly improved these negative changes.

The quantitative scoring in Table 5 confirmed the ameliorative effects of all co-treatments, specifically the niosomal forms, against ATZ-induced histological alteration of reproductive organs.

## Discussion

The extensive global use of ATZ ensures continued human and wildlife exposure to this herbicide and its metabolites, raising significant public and environmental health concerns. This is particularly critical for male reproductive health, as substantial in vitro and in vivo evidence confirms ATZ’s capacity to act as an endocrine disruptor^51^. This study was designed to evaluate and compare the efficacy of two antioxidants (LC and QT) in both their conventional and novel niosomal forms (LCLN and QTLN) for ameliorating ATZ-induced testicular toxicity.

Niosomal formulation of LC is properly made. Because smaller vesicles have a large surface area and high drug absorption, vesicle size is crucial for assessing niosome activity^52,53^. Herein, LCLN formulation showed a small particle size of 241.7 ± 1.5 nm with a low PDI, suggesting formation of nano-sized vesicles with uniform size distribution, reduced interfacial tension, and homogeneous vesicle formation. The PDI was used to quantify the extent of dispersion, distribution of size, and vesicle size uniformity within a sample^31^. Moreover, the prepared formulation possessed a negative surface charge, indicating good physical and chemical stability, enough for electrostatic stabilization. In this regard, Zeta potential, which gauges the charges that the vesicles have accumulated, can be used to evaluate the stability of colloidal dispersions^52^.

Additionally, optimized QT-loaded niosomes (QTLN) demonstrated effective preparation, achieving a small particle size of approximately 291.1 ± 5.2 nm with a low PDI, which signifies uniform size distribution, reduced interfacial tension, homogeneous vesicle formation, and minimized aggregation tendency^35^. The value of the zeta potential showed that the surface had a negative charge, which is good for electrostatic stabilization^35,54^. The negative charge in QTLN formulations generates electrostatic repulsions between vesicles, thereby enhancing vesicle stability while also promoting increased vesicle permeability^35,55^.

The preliminary studies were accomplished to determine the suitable type and concentration of non-ionic surfactants and cholesterol necessary for niosomes preparation with acceptable consistency for LC or QT delivery. When the amount of non-ionic surfactants and cholesterol increases, the amount of the hydrophobic domain and the number of niosomes produced raises and hence increases in entrapment efficiency and particle size^56,57^. However, increasing non-ionic surfactants and cholesterol beyond optimal levels (above 30 mg) led to reduced entrapment efficiency and larger particle sizes in niosomes, likely due to disrupted vesicular structures and potential formation of mixed micelles^56,57^. So, in the present study, 30 mg of non-ionic surfactants and cholesterol were used in the preparation of LCLN and QTLN.

According to the review of literature, different non-ionic surfactants such as Span 60 and Tween 60 in different molar ratios with cholesterol were studied to determine the optimum composition for LCLN or QTLN formulation. Span 60 niosomal formulations exhibited lower particle size when compared to Tween 60 niosomal formulations, with the same molar ratio with cholesterol. These results were obtained because of the length of the C-H alkyl chains^58^, low hydrophilic lipophilic balance (HLB) value and the hydrophobic moiety^59^, the higher the gel-liquid phase transition temperature and low surface free energy^57^ of Span 60. The presence of cholesterol in the niosomes formulation was considered important for keeping the assemblage integrity and physical stability and rigidity of these vesicles^60–62^. With Span 60 surfactant, the increase in the amount of incorporated cholesterol above the equi-molar ratio was accompanied by an increase in the bilayer membrane rigidity. Rigid membrane assembles as large vesicles, so an increase in the size of the prepared niosomes^63^. Higher amounts of cholesterol may compete with the drug to orientate space inside the bilayers leading to the exclusion of the drug from vesicles^64,65^. According to literature reviews, niosomes including 30 mg of Span 60 and cholesterol with 1: 1 molar ratio are efficient carriers for delivering drugs^64,65^. Therefore, Herein, LCLN and QTLN were formulated using 30 mg of Span 60 and cholesterol with 1: 1 molar ratio.

Male fertility is significantly reduced by oxidative stress. This is due to the fact that the testis is particularly susceptible to oxidative damage, owing to the plentiful cell division processes, elevated levels of oxygen consumption by the mitochondria within the testes, and higher concentrations of unsaturated fatty acids^66^. A useful stand-in for measuring lipid peroxidation in biological systems is the measurement of MDA^67^.

The present study observed that rats treated with ATZ alone had significantly higher blood MDA levels and lower SOD, GPx, and CAT levels than the control group. In agreement with our findings, ATZ treatment was observed to raise MDA level in the serum while TAC had significantly decreased compared to control group^68^. Also, ROS and MDA concentrations, which are markers of oxidative stress, were elevated after ATZ exposure^69^. Exposure to ATZ resulted in a dose-related drop in TAC, a reduction in GSH levels, and an increase in MDA content^67^. Levels of SOD were significantly decreased in blood, while MDA was elevated in ATZ-exposed *C. gariepinus*^70^. This was explained as ATZ causes oxidative stress by forming ROS^71^, lipid peroxidation and antioxidant depletion^72^. Following exposure to ATZ, the *Nrf/HO-1* pathway was increased at the mRNA level in the testis tissue, suggesting that ATZ has harmed rats’ reproductive systems through oxidative stress^73^.

Co-treatment with QT and LC, either niosomal or conventional, significantly improved oxidant-antioxidant markers. MDA levels decreased while SOD, GPx, and CAT levels increased compared to the ATZ group. Furthermore, QTLN and LCLN were found to attenuate ATZ-induced oxidative stress more effectively than their conventional counterparts. Consistent with our findings, LC greatly decreases the body’s reaction to oxidative stress and boosts the activity of the antioxidant system. After LC treatment, there was a notable decrease in MDA levels, suggesting a reduction in ROS production. Additionally, the levels of SOD and GPx, which are part of the enzymatic antioxidant system, showed an increase^74^. Administration of LC at 100 mg/kg with DIM (dimethoate) increased activities of SOD, GPx, and CAT, while it significantly decreased MDA levels and increased GSH levels^75^.

In addition, the niosomal form of LC offers greater protection than its conventional one. In this context, supplementing laying hens with L-carnitine-loaded solid lipid nanoparticles (LC-SLNs) has a beneficial antioxidant effect on their oxidative stress state, which may contribute to the enhanced cell permeability and availability of LC-SLNs over plain hydrophilic LC^19^, which confirmed our findings.

Regarding QT’s protective effects against oxidative stress, serum MDA levels were lower and levels of serum CAT were significantly greater in the groups treated with QT and nano QT in comparison to the 5-FU group^76^. In that study, oxidative stress markers improved even more significantly with nano QT delivery after 5-FU treatment than with QT treatment, which validated our findings.

Because of its phenolic group’s capacity to transfer a proton and stabilize free radicals, QT has the ability to scavenge free radicals. The excellent antioxidant activity of QT assists in protecting the human body from oxidative stress harm by preventing and reducing lipid, protein, and DNA oxidation in vivo^77^. The poor absorption of conventional QT limits its bioavailability and biological activity. On the other hand, the nanoparticle delivery system’s capacity to enhance the absorption and bioavailability of bioactive compounds has led to its widespread use^78^, thereby corroborating our premise that QTLN is more effective than QT in relieving oxidative stress.

The present investigation revealed that ATZ exposure significantly reduced the absolute and relative weights of the seminal vesicles. However, no significant alterations were observed in the weights of the testes, epididymis, or prostate gland compared to the control group. Our findings regarding the specific reduction in seminal vesicle weight align with previous research of Abarikwu et al.^79^ that reported a significant decrease in the absolute weight of the seminal vesicles in rats administered a high dose of ATZ (200 mg/kg BW), while finding no impact on testicular or epididymal weights. Similarly, Zhu et al.^73^ demonstrated that ATZ exposure (120 mg/kg) did not affect the relative weights of the testes but resulted in a reduction of absolute weights of accessory sex organs, including the seminal vesicles.

Disparities in the literature concerning ATZ’s effects on reproductive organ weights are evident, with some studies reporting reductions^80^ and others even reporting increases under certain dosing regimens^51^. This inconsistency is likely attributable to critical experimental variables such as species differences, dosage, exposure duration, and overall study design^73^. The specific reduction in seminal vesicle weight observed in our study may be linked to ATZ’s targeted interference with the pathophysiology and development of this specific accessory gland^81^.

Co-administration of LC and QT, in both conventional and niosomal forms, with ATZ resulted in a mild, non-significant recovery of the reduced seminal vesicle weights. The potential of LC to normalize reproductive organ weights has been previously documented; it protected against CuSO4-induced reductions in seminal vesicle weight, an effect attributed to its antioxidant properties that mitigate oxidative stress and support the structural integrity of reproductive tissues^82^. Likewise, QT has been shown to prevent lesions in the seminal vesicles of diabetic mice by mitigating oxidative stress^83^.

In this study, regarding reprotoxicity of ATZ, it worsened semen quality as well as reduced serum testosterone, FSH and LH levels when compared to control group. These findings were in agreement with previous findings in rats^73^ that detected a substantial reduction of sperm quality as compared to the control group. Moreover, reductions in intra-testicular testosterone, serum FSH, LH, and testosterone in rats were achieved after orally gavaging with ATZ alone (50 mg/kg)^84^. The primary reason for ATZ’s subpar sperm quality and serum hormonal levels is mostly due to the development of oxidative damage. Following ATZ exposure, an increase in MDA and a decrease in TAC were detected^85^, which corroborated our oxidative marker results. This may be explained by the high concentration of long-chain polyunsaturated fatty acids (PUFA) in the membrane of spermatozoa. The peroxides shorten the sperm’s life in vivo during fertilization or in vitro during laboratory preservation^86^. Also, peroxides cause significant alterations in sperm configuration, particularly in the acrosomal area, and a severe reduction in sperm motility. Oxidative stress may result in the reduction and degeneration of Leydig cells as well as a decline in their ability to synthesize testosterone, which is essential for optimal spermatogenesis^67^.

Atrazine also has negative effect on the pulsatile production of GnRH, which in turn results in lower levels of androgen. This reduces peak levels and surges of LH as well as FSH^87^. Furthermore, in our effort to shed light on the molecular mechanism underlying ATZ’s ability to lower serum levels of testosterone, FSH, and LH, we examined the expression of certain genes and discovered that exposure to ATZ significantly downregulated the relative expression levels *of 3β-HSD*,* StAR*,* and CYP11A1*, which may help to explain why exposure to ATZ lowers these hormone levels. In this context, by potentially interfering with the transcription of steroidogenic genes including *3β-HSD* and *17β-HSD*, ATZ may have an anti-androgenic impact^39^. These genes control the entire steroid-to-hormone conversion process by being linked to important enzymes that convert steroids into steroid hormones^88^, which corroborates our findings. In summary, the aforementioned information indicates that ATZ reduced the levels of FSH and LH hormones, which subsequently resulted in decreased testosterone levels, either through the induction of oxidative stress in the testes or by impairing steroidogenic genes, thereby disrupting spermatogenesis and compromising semen quality.

On the other side, our results clarified that co-administration of either LC, LCLN, QT or QTLN with ATZ dramatically improved semen quality and serum hormonal profile when compared to the ATZ group. Consistent with our findings, prior studies have demonstrated that LC enhances sperm parameters. Viability, morphology, and sperm counts are all directly enhanced^89^. The administration of LC improved serum free testosterone, total testosterone, LH, and FSH levels in buprenorphine-treated rats^90^. According to the positive impacts of LCLN, testosterone hormone levels significantly increased in rats receiving nano LC and lead acetate than in rats receiving lead acetate alone^91^.

The enhancing effects of LC or LCLN on semen quality and serum hormonal profile is mainly attributed to their antioxidant and androgenic properties^92^. The administration of LC inhibits the impairment of Leydig and Sertoli cells caused by oxidative stress^93^, which in turn resulted in increased hormonal levels of testosterone, FSH, and LH^94^. Also, after LC treatment, sperm motility and density were significantly increased in rats as LC is secreted as an antioxidant and accumulates in the epididymis, closely improving sperm motility and morphology^95^. These results corroborated our oxidative marker data, which similarly demonstrated a noteworthy amelioration with LC and LCLN against testicular oxidative challenges generated by ATZ. Another possible explanation is that LC works at the hypothalamic level; LC was found to exert a positive impact on the hypothalamic-pituitary-gonadal axis^96^. Also, our results about the gene expression analyses revealed that either LC or LCLN greatly upregulated the expression of *3β-HSD*, *StAR* and *CYP11A1* genes, thereby significantly reducing the deleterious effects of ATZ on decreasing such hormones. This could explain the genetic mechanism by which LC or LCLN enhances the studied hormones and subsequently improves spermatogenesis. In this context, the simultaneous administration of LC and lead acetate caused an increase in the levels of *17β-HSD* in the testes^89^, which is the primary enzyme found in the testis that transforms androstenedione into testosterone. Our results also could be explained in the light of the study of^68^, who found that LC was able to abolish the toxic effect of ATZ through the improvement of *CYP17A1* mRNA and consequently raising of serum testosterone^97^. Likewise, through the amelioration of testicular oxidative stress status, enhancement of steroidogenic enzyme activity, and elevation of *StAR* gene expression, nano LC mitigated CYP-induced decline in testicular hormone levels^98^. Testosterone together with FSH and LH play a crucial part in spermatogenesis, which enhances the quantity and quality of semen^99^. Therefore, the improving effects of LC and LCLN on these hormones is another possible mechanism to enhance semen quality deteriorated by ATZ.

The better effect of LCLN than LC was evidenced by the findings of earlier studies^92,100^, likely due to physical attributes (including shape, size, and zeta potential), alongside enhancements in encapsulation efficiency, drug content, drug loading capacity, drug release, and in vitro release of the formulated LC-SLNs, all of which were optimized in the niosomal form^19^.

Concerning QT and QTLN, in line with our findings, they improved semen quality of ATZ-exposed rats^101^. In a 70-day rat study, serum levels of LH and testosterone were considerably elevated when oral QT in a dosage of 50 mg/kg body weight was delivered concurrently with fenitrothion, as opposed to fenitrothion alone^102^. Concerning the improving effects of QTLN, it has been found that when rooster sperm were stored at 4 degrees for 24 and 48 h, QT in the form of nano-liposomes dramatically increased the parameters of sperm total motility, progressive motility, viability, and integrity of sperm membrane in comparison to the control group^103^. This is attributed to the potential antioxidant activity of QT or QTLN. QT has been reported to mitigate the detrimental impacts of oxygen radicals, enhance the functional characteristics of spermatozoa, decrease lipid peroxidation levels, and augment antioxidant properties in rats^104^. Another reason is due to that QT revealed a protective action against testicular toxicity caused by cypermethrin and deltamethrin by controlling the levels of enzymes linked to steroidogenesis (*3β-HSD and 17β-HSD*) in rats’ testes and further bringing testosterone levels back to normal^105^. In addition, our data of gene analysis proved for the first time, to the best of our knowledge, that QTLN could improve testosterone, FSH, and LH hormones that were reduced by ATZ by upregulation of mRNA expression *of 3β-HSD*,* CYP11A1 and StAR genes*.

Furthermore, in the present study, the improving activity on sperm quality and hormonal profile for QTLN was observed to be significantly greater than conventional form. This is because the bioavailability and biological activity of conventional QT is limited. Since the nanoparticle delivery system can increase the absorption and bioavailability of bioactive substances, it has been widely employed for bioactive chemicals whose low absorption limits their biological activity and bioavailability^78^. This is also attributed to the decreased crystallinity, higher solubility, higher probability of permeability to tissues and increased stability of nano QT than its conventional form^106^.

In the current study, ATZ significantly reduced the mRNA relative expression levels of *3β-HSD*,* StAR*,* and CYP11A1* in testes. The process of steroidogenesis begins with the transport of cholesterol into the mitochondria via *StAR* gene. There, it is absorbed by *CYP11A1* and subsequently converted to pregnenolone. *3β-HSD* converts pregnenolone to progesterone, whereas *CYP17A1* catalyzes the conversion of progesterone to androstenedione^107^. According to Kolaitis et al.^10^, steroidogenic gene transcript levels in the mouse testis can be changed as a result of long-term exposure to an ambient ATZ concentration. Exposure to ATZ reduces the mRNA expression of steroidogenic genes (*StAR*,* CYP17A1*,* and 17β-HSD*) which is directly linked to decreased androgenesis, thus, impairs the steroidogenesis of Leydig cells^108^. Additionally, in the testes of animals treated with ATZ, there was a decrease in the immunoexpression of *3β-HSD* compared to the control animals^8^.

The most remarkable finding in the current study, is that all treatments (LC, LCLN, QT, and QTLN) significantly upregulated the expression of all studied steroidogenic genes, thereby significantly reducing the deleterious effects of ATZ on steroidogenesis. Consistent with our findings, treatment with LC substantially raised the expressions of *STAR* and *CYP11A1* in male rats exposed to Monosodium glutamate. Hence, LC may cause the serum level of testosterone to rise noticeably^96^. According to a previous investigation, LC administration (500 mg/kg) overexpressed the testicular *StAR* gene^109^.

While there is little information available about the improving genetic effect of LCLN, a previous study^98^ found that nano LC could improve the activity of testicular steroidogenesis enzymes (*3β-HSD* and *17β-HSD*) and upregulate the expression of the *STAR* gene. These factors are likely to be responsible for the concurrent improvements in sperm properties and sex hormone levels of testosterone, FSH, and LH. It is widely recognized that oxidative stress and the production of ROS have the potential to hinder mitochondrial activity^110^, leading to the suppression of *STAR* gene expression. This elucidates the manner in which LCLN exerts a beneficial influence on the gene’s expression, owing to its inherent antioxidant characteristics.

Concerning QT effects, a previous research suggested that QT reduces pyrethroid-induced reproductive toxicity and oxidative damage in rats by increasing the activities of *3β-HSD* and *17β-HSD*, leading to increased testosterone levels. The explanation for that could be due to the noticeable increase in oxidative stress in the testicular tissue, causing Leydig cell destruction. Thus, the administration of QT, which has potent antioxidant activity, to rats resulted in an increase in steroidogenic enzymes (*3ß-HSD and 17ß-HSD*), which in turn enhanced the levels of sex hormones (FSH, LH, and testosterone) and the quality of semen^105^. According to Abarikwu et al.^111^, QT (10–50 µM) was adequate to increase *CYP11A1* promoter activity. By obstructing the COX2-dependent signaling, QT has also been demonstrated to increase *STAR* gene expression and steroidogenesis in Leydig cells^112^.

Information about the beneficial effects of QTLN on steroidogenic genes is scarce, in this respect, nano QT co-exposure with triazophos brought back the levels of steroidogenic enzyme (*3β-HSD )* in testicular cells in comparison with the triazophos high-dose group^106^. Niosomal quercetin’s superior activity over QT is primarily related to its capacity to increase the absorption and bioavailability of these compounds^78^, which fortified our findings.

In summary, based on the findings presented above, our study shed insight on the molecular mechanism by which LC or QT, whether in conventional or niosomal form, has a good impact on male fertility. We assume that both treatments, notably the niosomal forms, significantly succeeded in protecting against ATZ-induced steroidogenic machinery disruption through overexpression of *3β-HSD*,* STAR*, and *CYP11A1* in the testes.

It was clear that ATZ induced degenerative histological changes in testicular, epididymal, seminal vesicular gland, and prostatic tissues. The histopathological quantitative scoring confirmed the degenerative effects of ATZ. Consistent with our findings, seminiferous tubules were destroyed as a result of ATZ treatment, and the germinal epithelium was separated from the basement membranes. The seminiferous tubules also lacked sperm in the lumen and contained a large number of vacuolations, uneven basement membranes, and few germinal cells. There were only a few Leydig cells that were highly stained, and there was acidophilic material buildup in the interstitial spaces^113^. The conceivable explanation for the negative histopathological effects of ATZ could be due to oxidative stress triggered by it^114^. Atrazine’s ability to cross the blood-testis barrier and obstruct the connection between Sertoli cells and germ cells provides an additional rationale for its negative histopathological effects.

Interestingly, co-treatment with LC, LCLN, QT, and QTLN significantly ameliorated all of these negative changes. Furthermore, the ameliorative effects were obvious in the niosomal groups compared to the conventional ones. The quantitative scoring confirmed the ameliorative effects of all co-treatments specifically the niosomal forms against ATZ-induced histological alteration of sex organs. Consistent with our findings, prior histological studies indicated that LC effectively alleviated the detrimental effects of methotrexate on the testes^115^. Furthermore, it was discovered by Baltaci et al.^116^ that administering QT to adult rats significantly reduced the degenerative alterations in seminiferous tubule structure and cell death caused by arsenic. These findings could be explained by the putative antioxidant activity of LC and QT^68^. As a result, it is conceivable to interpret the powerful antioxidant capabilities of both treatments, which scavenge produced free radicals and prevent lipid peroxidation while keeping normal testicular architecture, as the manner that QT and LC protect against ATZ-induced testicular degeneration^117^.

In addition, the better effects of LCLN or QTLN than their conventional form may contribute to the enhanced cell permeability and availability^19,118^, which confirmed our findings.

The superior efficacy of niosomal formulations over conventional forms can be attributed to several inherent advantages of niosomes as vesicular drug delivery systems. Niosomes are self-assembled nanovesicles composed of non-ionic surfactants and cholesterol that enhance the pharmacokinetic profile of encapsulated drugs through multiple mechanisms [122]. First, niosomes improve the solubility and bioavailability of poorly water-soluble compounds such as QT, while also protecting labile drugs like LC from premature degradation in the gastrointestinal tract [122,123]. Second, the niosomal bilayer structure enables controlled and sustained drug release, as the vesicles gradually release their cargo over an extended period rather than releasing it all at once [122,124]. This controlled release was demonstrated in a comparative study where niosomes maintained drug release for over 24 h, whereas conventional liposomes released their payload within 4 h upon enzymatic exposure [124]. Third, niosomes facilitate targeted delivery to specific tissues (testicular tissue) by improving drug absorption and prolonging circulation time, thereby enhancing drug accumulation at the target site [122,125]. Additionally, the enhanced stability of niosomes compared to other vesicular carriers, due to the chemical stability of non-ionic surfactants against oxidation and hydrolysis, ensures that more of the administered dose reaches the systemic circulation intact [122,123]. Collectively, these pharmacokinetic and biopharmaceutical advantages explain why niosomal encapsulation of LC and QT produced greater restoration of sperm quality and testicular integrity compared to their conventional forms.

## Conclusion

Our study revealed that ATZ, as a prevalent environmental toxicant, induced a significant lowering of sperm quality and serum male sex hormone, which was primarily caused by oxidative stress induced by ATZ, as demonstrated by lower serum SOD, CAT, and GPx levels and higher serum MDA. ATZ inhibited steroidogenesis by lowering FSH, LH, and testosterone levels, and we discovered that ATZ dramatically reduced the mRNA expression of certain steroidogenic genes (*Cyp11A1*,* HSD-3b*,* and StAR*) in the testes. Histopathological examination and lesion scoring data, all proved ATZ’s negative effects on testicular function. Intriguingly, combining LC, LCLN, QT, or QTLN with ATZ greatly mitigated all of its toxic effects. Furthermore, the niosomal form of both treatments produced superior results than the conventional form.

Furthermore, a key limitation of this study is the absence of in-depth exploration into the molecular mechanisms underlying the niosomal formulations of both treatments (QTLN and QTLN), particularly how they enhance male fertility by modulating specific genetic pathways such as Nrf2/HO-1, sperm DNA damage (include assays such as Sperm Chromatin Dispersion (SCD) test or Comet assay for sperm DNA integrity) as well as the lack of evaluation regarding their potential applicability in humans.

## Supplementary Information

Below is the link to the electronic supplementary material.

Supplementary Material 1

## Data Availability

The data that support the findings of this study are available upon reasonable request.
