# Supplementary material for: Niosomal l-carnitine and quercetin improve sperm quality and testicular function in atrazine-induced reproductive toxicity in rats
Source: Sci Rep. 2026 Jun 22;16:19358. doi: 10.1038/s41598-026-58143-4 (PMC13287762; doi:10.1038/s41598-026-58143-4)
Supplement: Supplementary file 1 — Supplementary Material 1 [file 41598_2026_58143_MOESM1_ESM.docx]

**Table S1: Fold changes of ATZ in relation to control.**

| Parameter | Control | ATZ |
| --- | --- | --- |
| Sperm motility (%) | 1 | 2.97↓ |
| Sperm count (Millions/ml) | 1 | 3.06↓ |
| Sperm morphology (Abnormal %) | 1 | 5.67↑ |
| Sperm viability (Viable sperm %) | 1 | 2.97↓ |
| Testosterone (ng/ml) | 1 | 7.13↓ |
| FSH (mIU/ml) | 1 | 7.01↓ |
| LH (mIU/ml) | 1 | 7.02↓ |
| MDA (nmol/ml) | 1 | 3.99↑ |
| SOD (U/ml) | 1 | 3.43↓ |
| GPx (U/L) | 1 | 1.98↓ |
| CAT (U/L) | 1 | 3.67↓ |
| 3β-HSD  mRNA relative expression level | 1 | 4.17↓ |
| StAR  mRNA relative expression level | 1 | 3.03↓ |
| CYP11A1  mRNA relative expression level | 1 | 3.23↓ |

↓ means that the values are decreased compared to the control.

↑means that the values are increased compared to control.

**Table S2: Fold changes of the study treatments in relation to ATZ**

| Parameter | ATZ | ATZ+LC | ATZ+LCLN | ATZ+QT | ATZ+QTLN |
| --- | --- | --- | --- | --- | --- |
| Sperm motility (%) | 1 | 1.36↑ | 1.89↑ | 1.51↑ | 1.83↑ |
| Sperm count (Millions/ml) | 1 | 1.65↑ | 2.28↑ | 1.79↑ | 2.38↑ |
| Sperm morphology (Abnormal %) | 1 | 1.41↓ | 1.59↓ | 1.88↓ | 2.28↓ |
| Sperm viability (Viable sperm %) | 1 | 1.47↑ | 1.97↑ | 1.58↑ | 1.90↑ |
| Testosterone (ng/ml) | 1 | 3.16↑ | 3.96↑ | 2.68↑ | 3.27↑ |
| FSH (mIU/ml) | 1 | 3.12↑ | 3.93↑ | 2.74↑ | 3.01↑ |
| LH (mIU/ml) | 1 | 3.12↑ | 3.92↑ | 2.73↑ | 3.01↑ |
| MDA (nmol/ml) | 1 | 1.39↓ | 1.69↓ | 1.41↓ | 1.72↓ |
| SOD (U/ml) | 1 | 1.92↑ | 2.51↑ | 1.93↑ | 2.54↑ |
| GPx (U/L) | 1 | 1.20↑ | 1.52↑ | 1.36↑ | 1.54↑ |
| CAT (U/L) | 1 | 2.25↑ | 2.52↑ | 2.28↑ | 2.67↑ |
| 3β-HSD  mRNA relative expression level | 1 | 1.92↑ | 1.71↑ | 2.33↑ | 2.83↑ |
| StAR  mRNA relative expression level | 1 | 1.48↑ | 1.67↑ | 1.91↑ | 2.21↑ |
| CYP11A1  mRNA relative expression level | 1 | 1.58↑ | 1.77↑ | 2.35↑ | 2.55↑ |

↓ means that the values are decreased compared to ATZ.

↑means that the values are increased compared to ATZ.


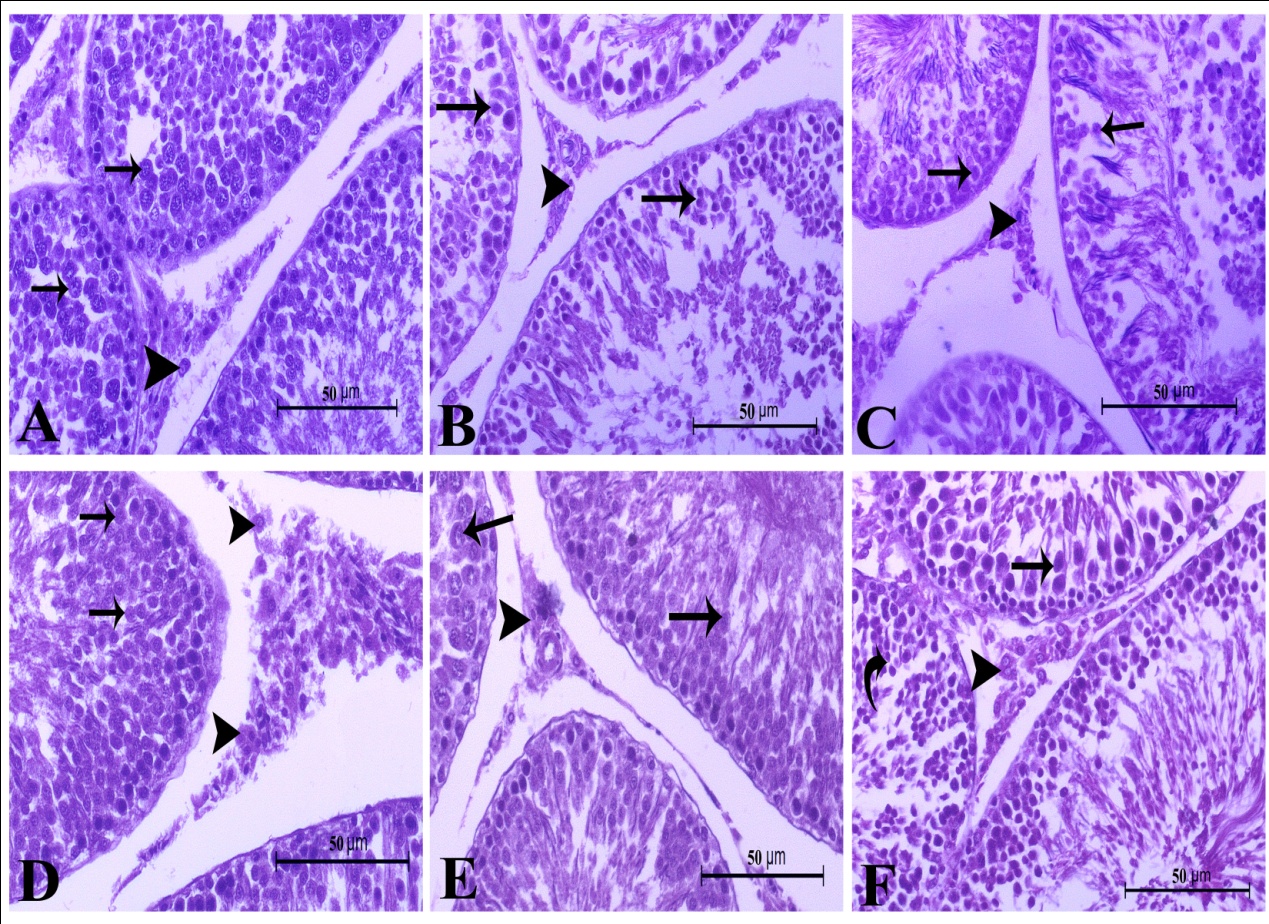


**Fig. S1: A higher magnification** of **testicular tissues in adult male albino rats of the studied groups showing:**

A: control group showed higher magnification of testicular tissue, including normal spermatogenic cells (arrow). The Leydig cells (arrowhead) appeared active.

B: Atrazine-treated group showed higher magnification of seminiferous tubules with degenerated spermatogenic cells (arrow). The interstitial tissue showed marked inactive Leydig cells (arrowhead).

C: Atrazine + L-carnitine group showed higher magnification of testicular tissue including more or less normal spermatogenic cells (arrow). The Leydig cells (arrowhead) appeared less active.

D: Atrazine + L-carnitine-loaded niosomes group showed higher magnification of testicular tissue including normal spermatogenic cells (arrow). The Leydig cells (arrowhead) appeared active.

E: Atrazine + quercetin-treated group showed higher magnification of testicular tissue including normal spermatogenic cells (arrow). The Leydig cells (arrowhead) appeared less active.

F: Atrazine + quercetin-loaded niosomes -treated group showed higher magnification of testicular tissue including normal spermatogenic cells (arrow). The Leydig cells (arrowhead) appeared active. (H&E stain X400).


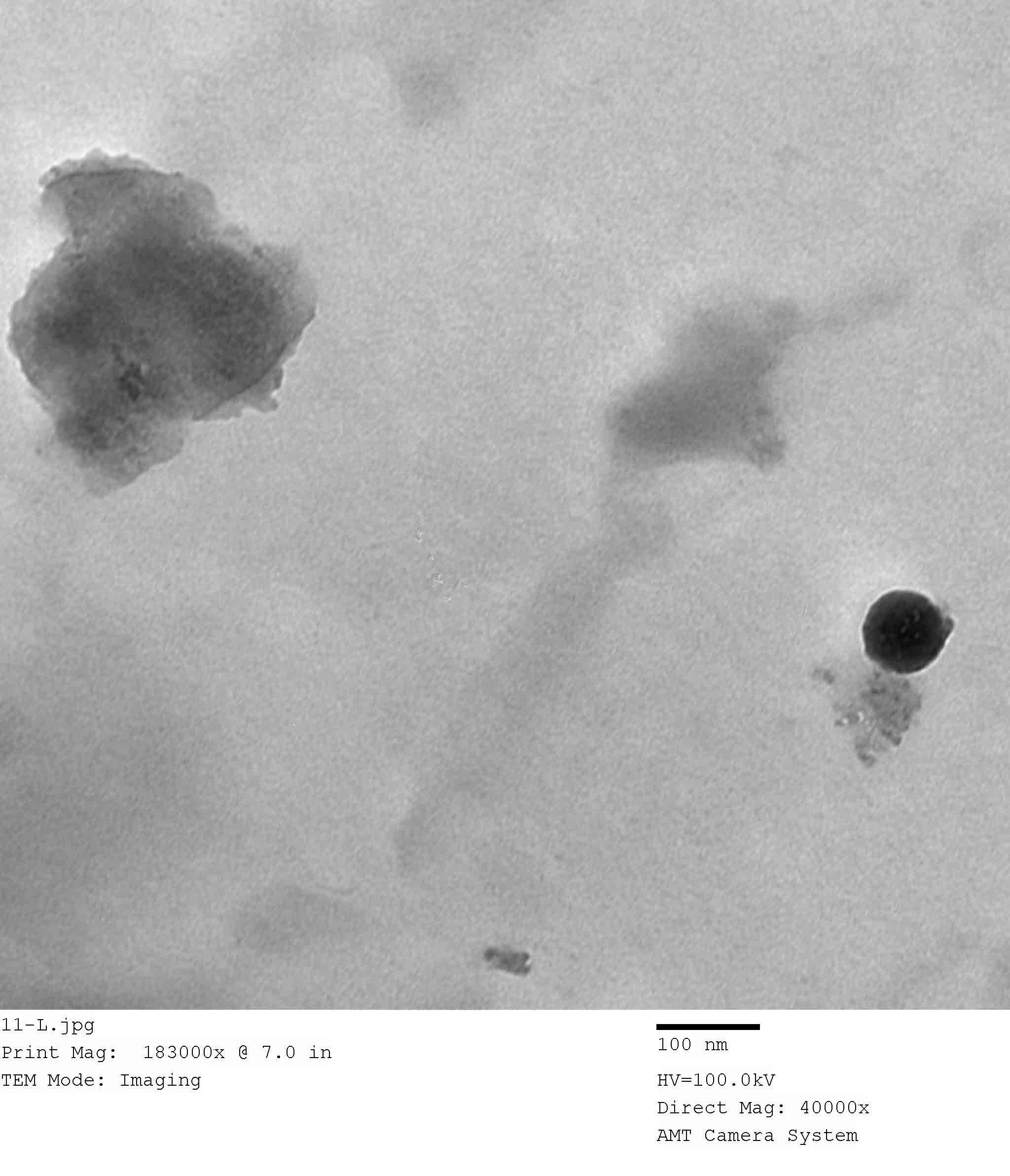


**Fig. S2: Surface morphology of L-carnitine-loaded niosomes formulation by TEM (100nm).**


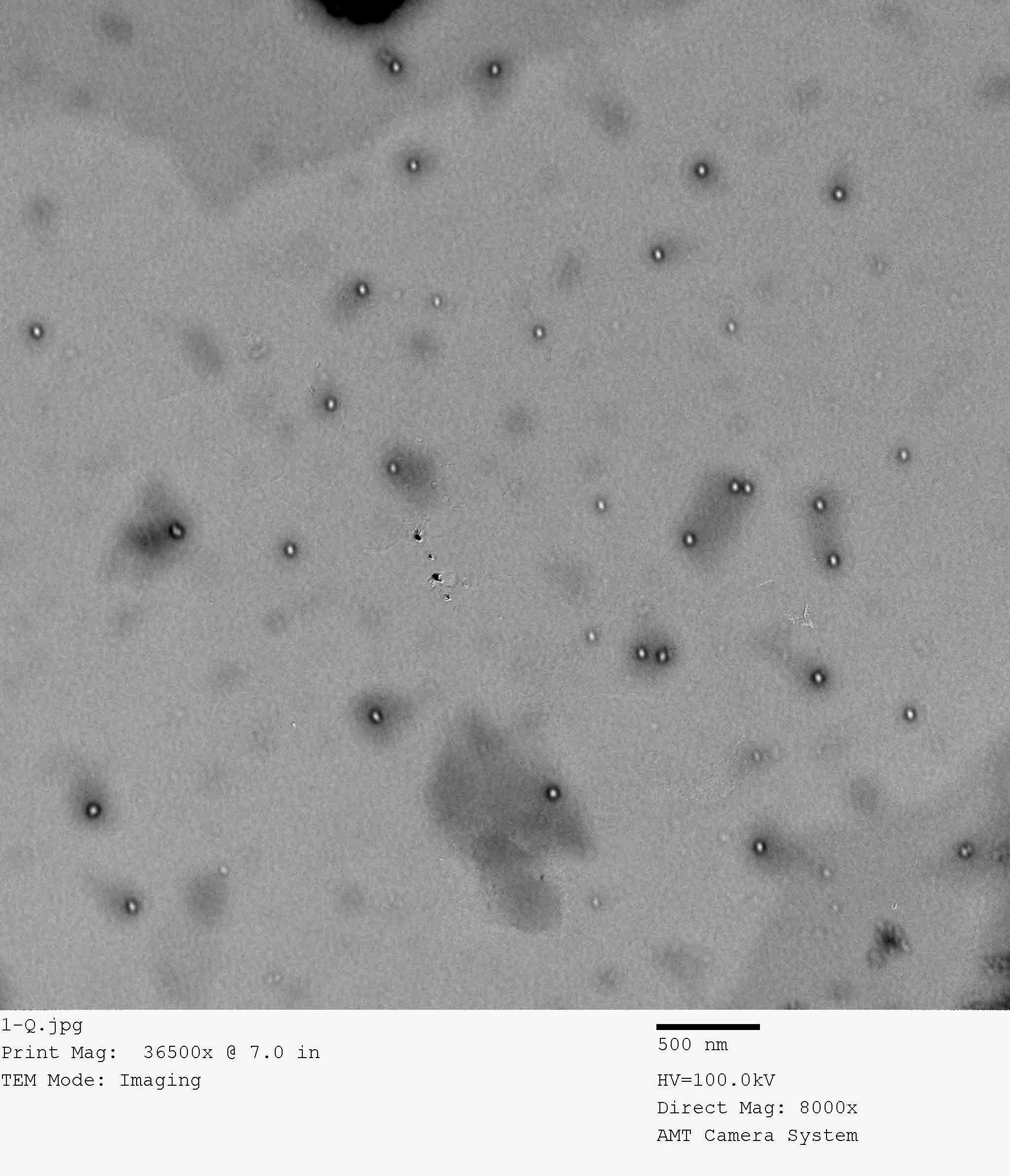


**Fig. S3: TEM image of optimum quercetin-loaded niosomes formulation (500nm).**


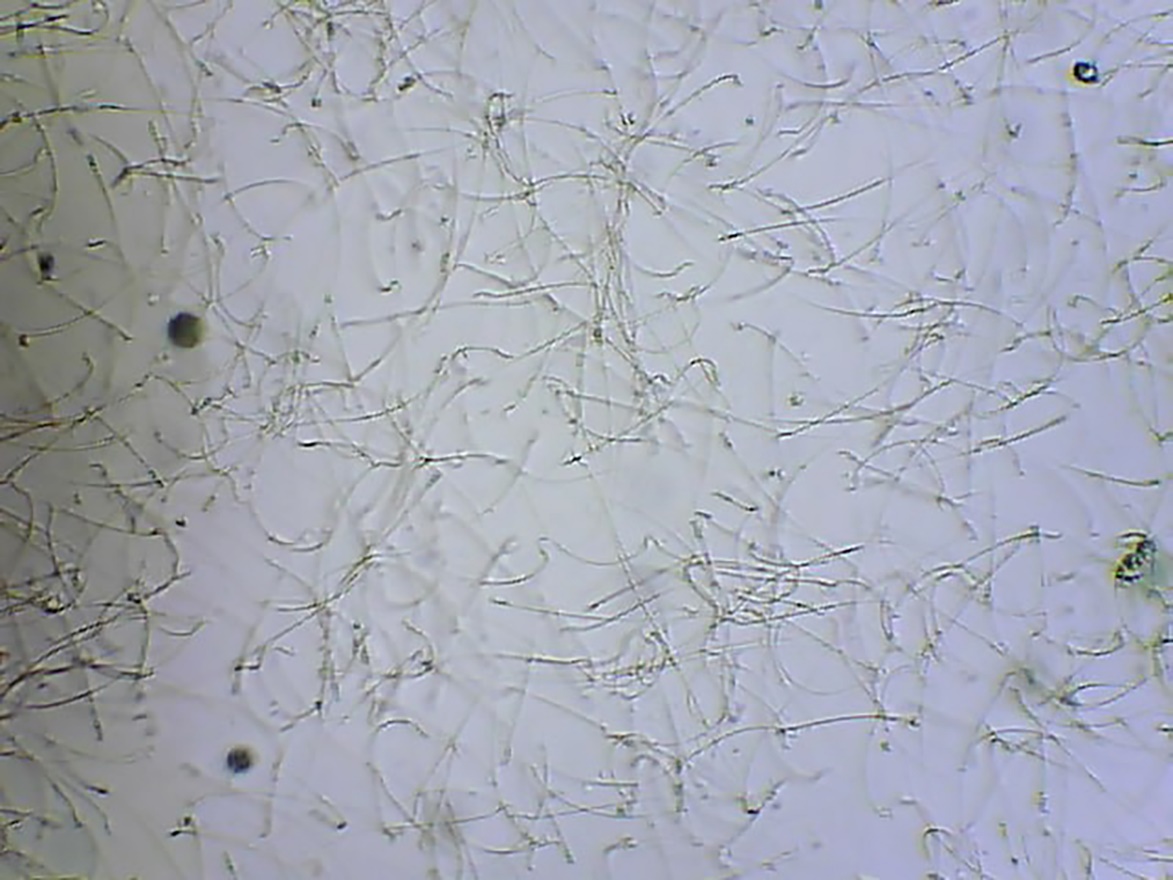


**Fig. S4: Image of rat semen sample of control group using CASA system.**


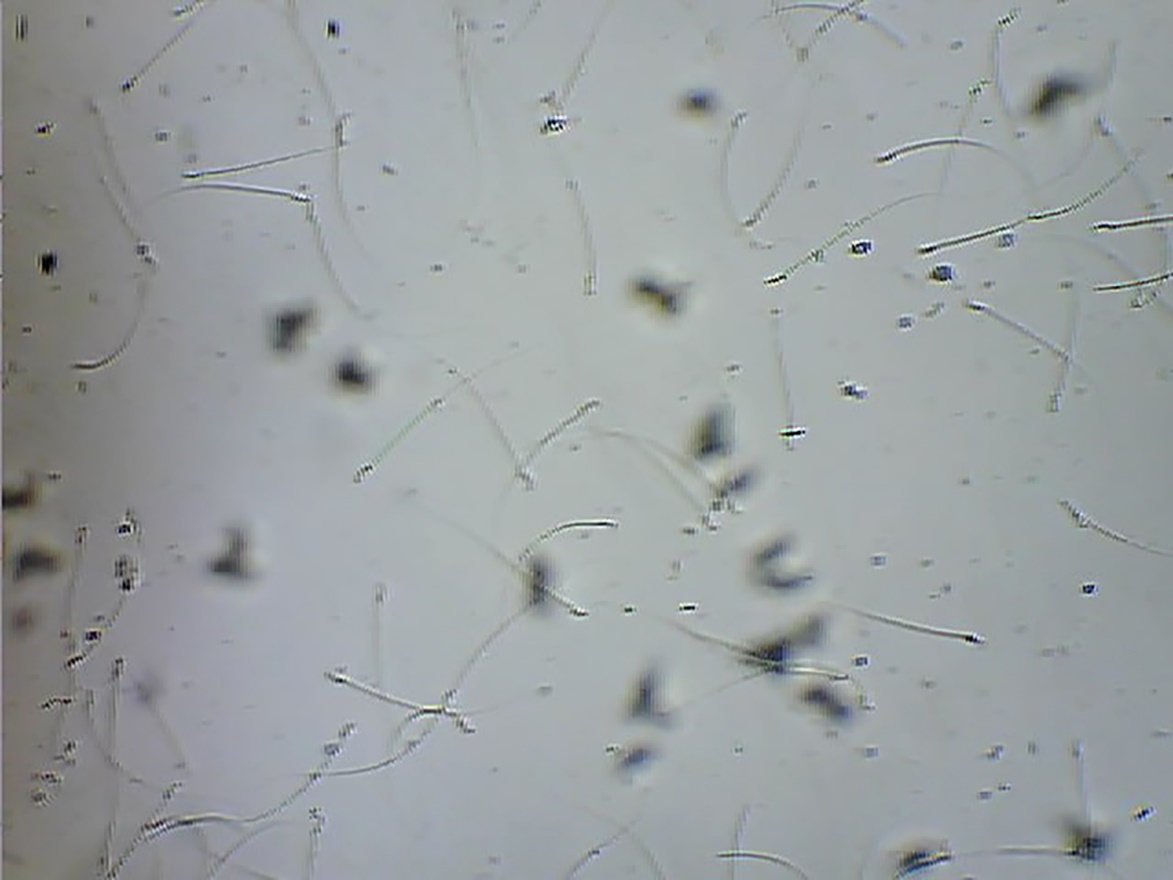


**Fig. S5: Image of rat semen sample of ATZ group using CASA system.**

**
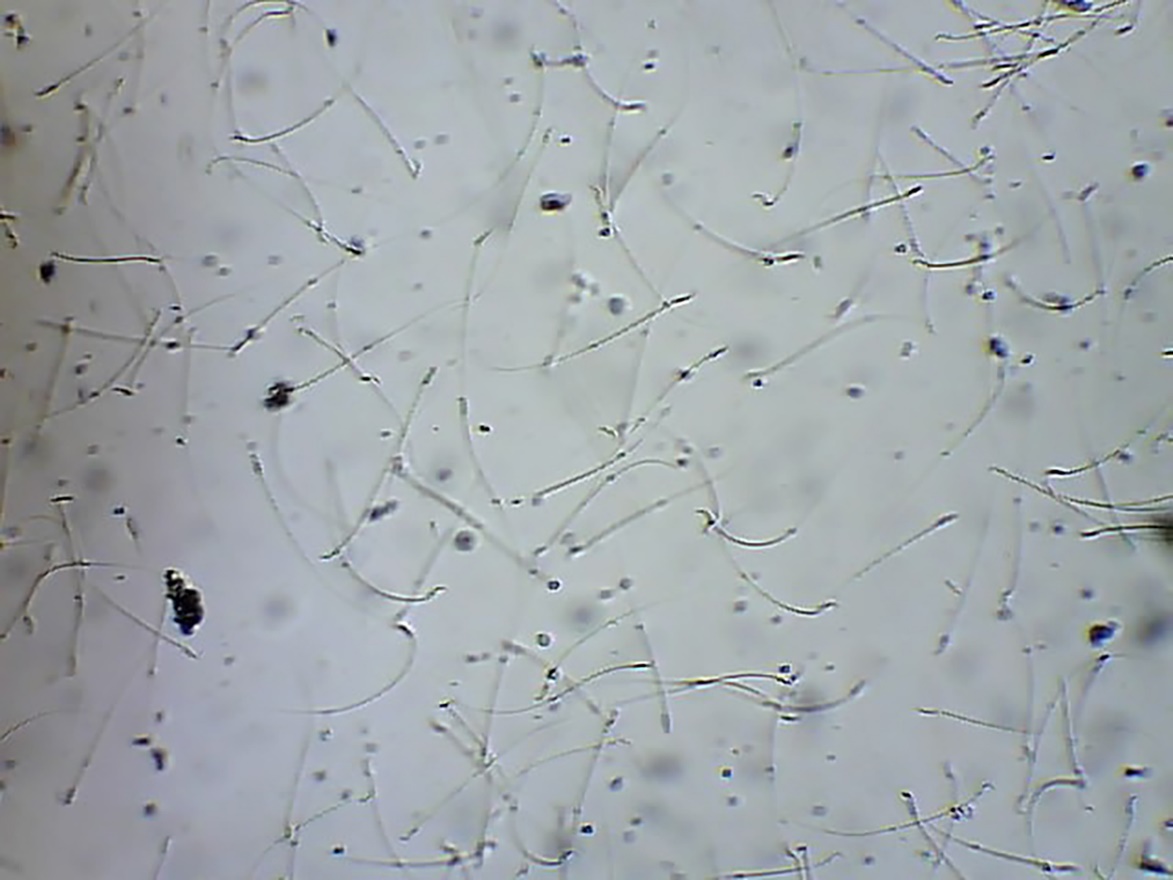
**

**Fig. S6: Image of rat semen sample of LC group using CASA system.**

**
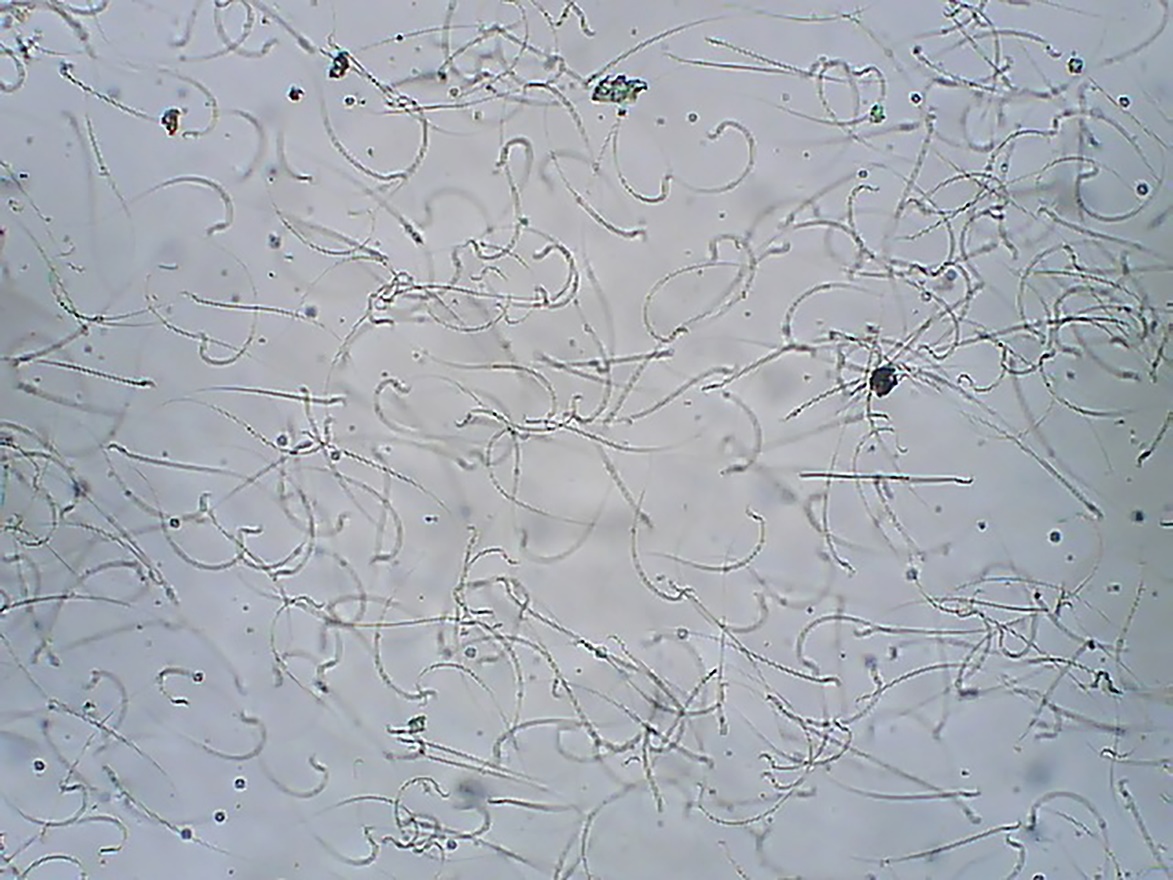
**

**Fig. S7: Image of rat semen sample of LCLN group using CASA system.**


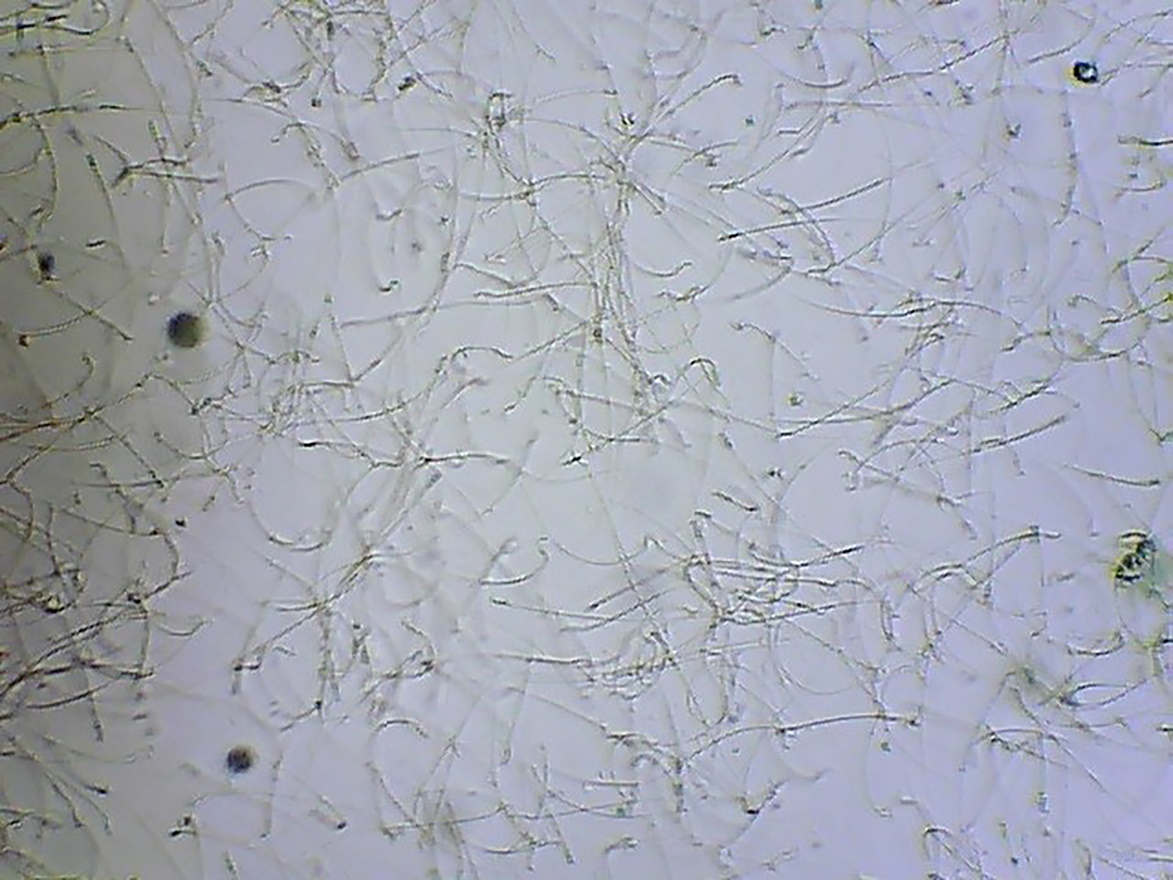


**Fig. S8: Image of rat semen sample of QT group using CASA system.**


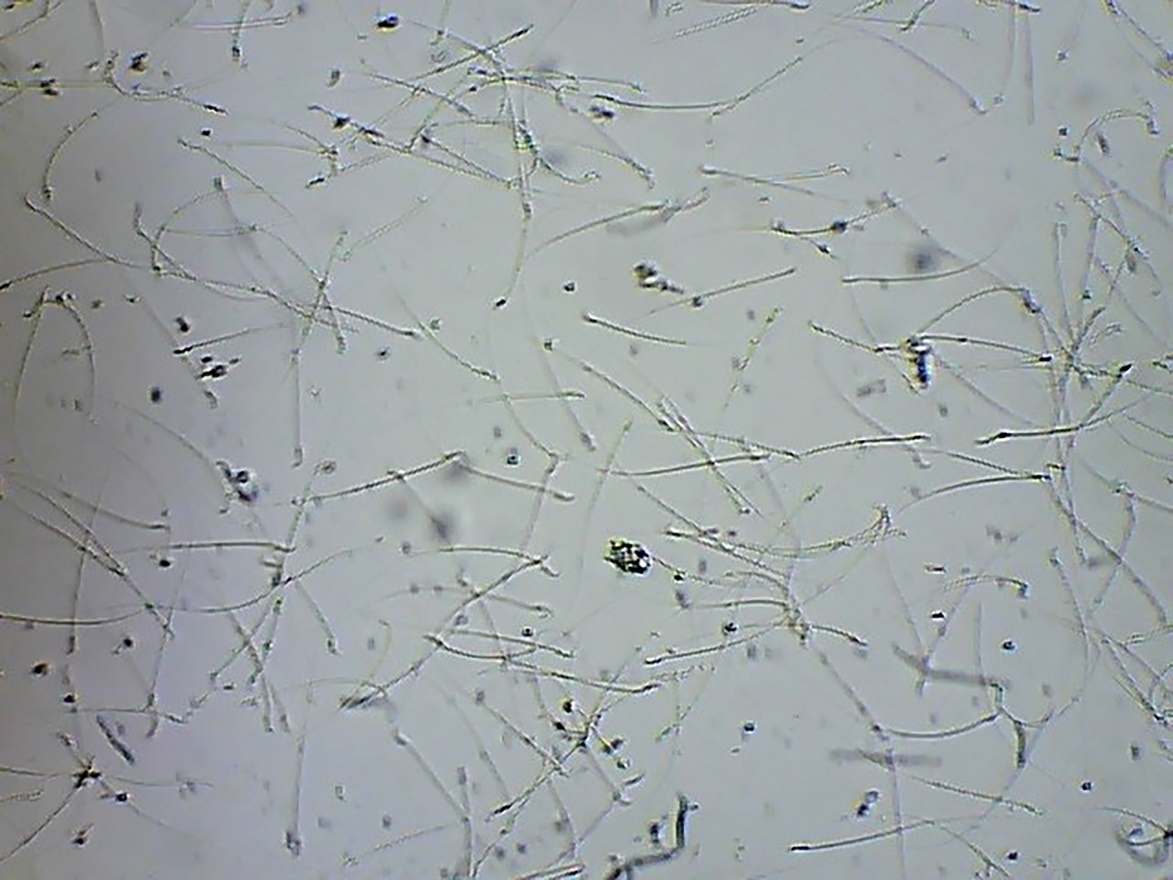


**Fig. S9: Image of rat semen sample of QTLN group using CASA system.**
